# Brønsted acid catalyzed remote C6 functionalization of 2,3-disubstituted indoles with β,γ-unsaturated α-ketoester

**DOI:** 10.3389/fchem.2022.992398

**Published:** 2022-09-13

**Authors:** You-Ya Zhang, Lin Li, Xiang-Zhi Zhang, Jin-Bao Peng

**Affiliations:** School of Biotechnology and Health Sciences, Wuyi University, Jiangmen, China

**Keywords:** remote C6 functionalization of indoles, β,γ-unsaturated α-ketoester, brønsted acid, metal-free, 2,3-disubstituted indoles

## Abstract

A metal-free catalytic approach for the remote C6-functionalization of 2,3-disubstituted indoles has been developed. In the presence of catalytic amounts of Brønsted acid, the *β,γ*-unsaturated *α*-ketoesters react with 2,3-disubstituted indoles at the C6 position selectively. Under mild reaction conditions, a range of C6-functionalized indoles were prepared with good yields and excellent regioselectivity. This methodology provides a concise and efficient route for the synthesis of C6-functionalized indole derivatives.

## Graphical Abstract

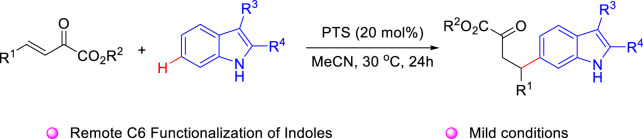

## Introduction

**SCHEME 1:**
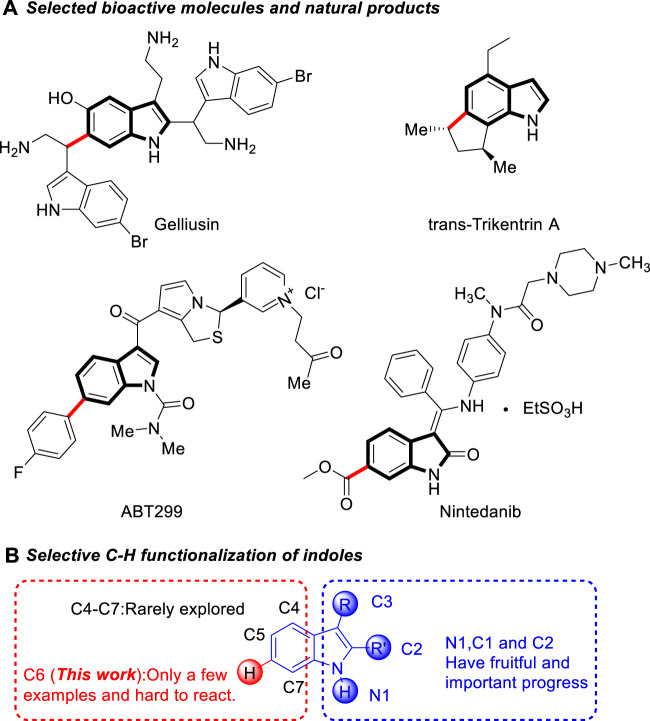
Selected bioactive molecules and natural products containing indole skeleton and selective C-H functionalization of indoles.

Indole and its homologues and derivatives widely exist in nature, mainly in natural flower oils, such as jasmine, bitter orange flower, daffodil, vanilla, etc. This structure also ubiquitously exists as key structural framework in numerous natural products, functional materials, and medicines (Ishikura et al., 2015; Sherer and Snape, 2015; Zhang et al., 2015; Sravanthi and Manju, 2016; Gao et al., 2020) (Scheme 1A). For example, tryptophan is an essential amino acid of animals. Trikentrins, which have a fused cyclopenta[*g*]indole structures, were isolated from sponges and showed antimicrobial activity and cytotoxicity against KB cells (Capon et al., 1986; Herb et al., 1990). ABT-299 is a prodrug that is highly potent and specific platelet activating factor (PAF) antagonist (Vaden et al., 1996). Nintedanib is an oral tyrosine kinase inhibitor approved for the treatment of idiopathic pulmonary fibrosis (Wind et al., 2019).

Due to their important biological activities, the synthesis of functionalized indoles has received continuous attention. Although traditional methods for the indole synthesis can lead to indoles with different functionalizations (Fischer and Jourdan, 1883; Sugasawa et al., 1979; Gassman et al., 1984; Street et al., 1993; Fukukyama et al., 1994; Furstner and Ernst, 1995) these methods require multi-step substrate synthesis and the introduction of functional groups in many cases are less efficient. An alternative approach to access functionalized indoles is the direct C-H functionalization of indoles, (Wang et al., 2016; Fan et al., 2017; Gandeepan et al., 2019; Rej et al., 2020), which allows for the rapid and efficient introduction of specific groups into the indole backbone, thus providing a most straightforward and atom-economical access to the target indoles. Numerous efforts have been made in recent years to achieve selective functionalization of indoles. One of the major challenges of this area is the controlling of the site-selectivity of indole (Cacchi and Fabrizi, 2005; Humphrey and Kuethe, 2006; Sandtorv, 2015; Yang and Shi, 2018). Due to its inherent nucleophilic characteristics, the reaction of indoles usually take place at their very reactive N1, C3 and C2 positions. (Scheme 1B) various methods have been developed for the C3, C2, and N1-functionalization of indoles *via* organo- and transition-metal-catalysis.

**SCHEME 2:**
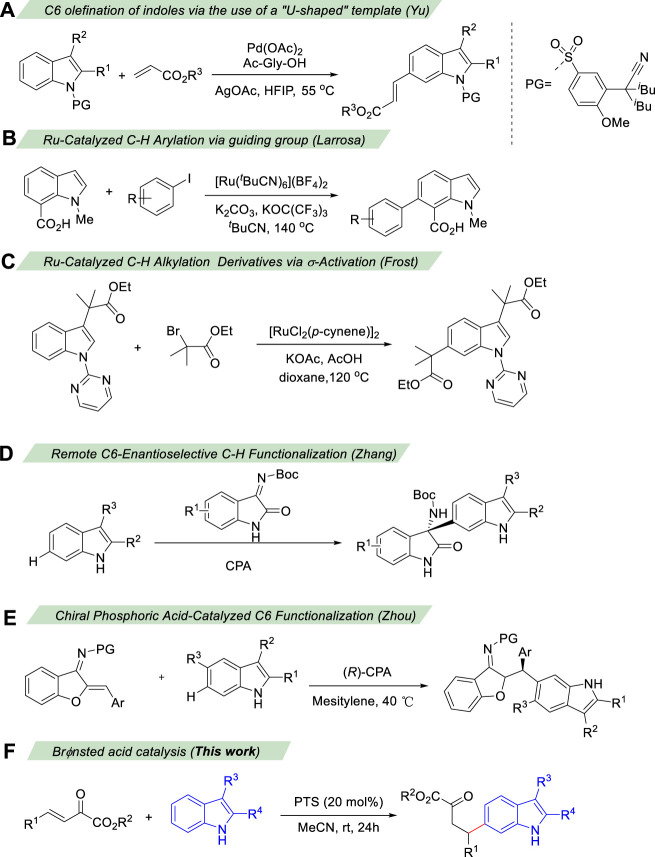
Transition-Metal Catalyzed C6 selective C−H Functionalization of Indoles.

Comparatively, the functionalization at the C4−C7 position of indoles has been less reported, most of them were based on transition metal catalysis using directing groups to achieve C-H bond activation (Poulsen et al., 2015; Rostoll-Berenguer et al., 2018; Xiao et al., 2018; Huang et al., 2019; Wen and Shi, 2021). Among them, the C6 position of indole is far away from the possible directing group, which makes the C6 functionalization of indole even more difficult (Liu et al., 2014; Zhou et al., 2014; Wu et al., 2019; Ling et al., 2019; Yan et al., 2020). In 2014, Yu and co-workers developed a removable “U-shaped” template to enable the orientation of indole C6-H to carry out alkenylation reactions (Yang et al., 2014) (Scheme 2A). In 2016, Larrosa developed a new strategy for indole C7 carboxyl group as a guiding group, causing indole C6 to undergo arylation (Scheme 2B). Simonetti et al. (2017) Frost’s group used C3 ester groups with auxiliary coordination orientation and strong coordination orientation of pyrimidine groups on N atoms as the reaction substrate, and achieved selective C-H bond alkylation of indole C6 in Ru catalysis (Scheme 2C). (Leitch et al. (2017) Recently, the groups of Zhang (Zhou et al., 2019) (Scheme 2D) and Zhou (Huang et al., 2021) (Scheme 2E) have independently developed C6-enantioselective C−H functionalization of 2,3-disubstituted indoles *via* chiral phosphoric acid catalysis. However, the development of efficient methods for the facile access to metal-free and highly selective C6 functionalization of indole in a sustainable fashion under mild conditions is still highly desirable. Herein, we developed a Brønsted acid catalyzed remote C6 functionalization of 2,3-disubstituted indoles with *β,γ*-unsaturated *α*-ketoester (Scheme 2F).

Initially, we examined this indole C6 functionalization reaction using *β,γ*-unsaturated *α*-ketoester 1a and 2,3-dimethyl-1*H*-indole **2a** as model substrates. To our delight, when a mixture of **1a** and **2a** in toluene were treated with benzenesulfonic acid (BSA) at 30°C, the expected reaction proceeds smoothly and produced the desired C6 functionalized product **3aa** in 42% yield (Table 1, entry 1). Inspired by this exciting result, we studied different reaction parameters for this reaction. Firstly, we screened the acid catalysts, including both Brønsted acids and Lewis acids (Table 1, entries 2–7). When *p*-toluenesulfonic acid (PTS) was used as the catalyst, the product **3aa** could be obtained in a yield of 54% (Table 1, entry 2). Subsequently, different solvents were examined. DCM and DCE provided the desired product **3aa** in moderate yields (Table 1, entries ht and 9). Other solvents such as THF and DMSO were ineffective for this reaction and only trace amounts of product were detected (Table 1, entries 10 and 11). MeCN was found to be the optimal solvent and the product **3aa** was obtained in 85% yield (Table 1, entry 12). Then, the influence of the reaction temperature to this transformation was investigated. When the reaction was performed at 0°C, the yield of **3aa** was decreased to 69% (Table1, entry 14). However, higher temperature also led to decreased yields (Table 1, entries 15 and 16). Finally, the ratio of the starting materials was screened (Table 1, entries 16–20). When 1.5 equivalent of 2,3-dimethyl indole was used, the desired product was obtained in an excellent yield of 92% (Table 1, entry 18).

**TABLE 1:** Optimization of the reaction conditions^*a*^.

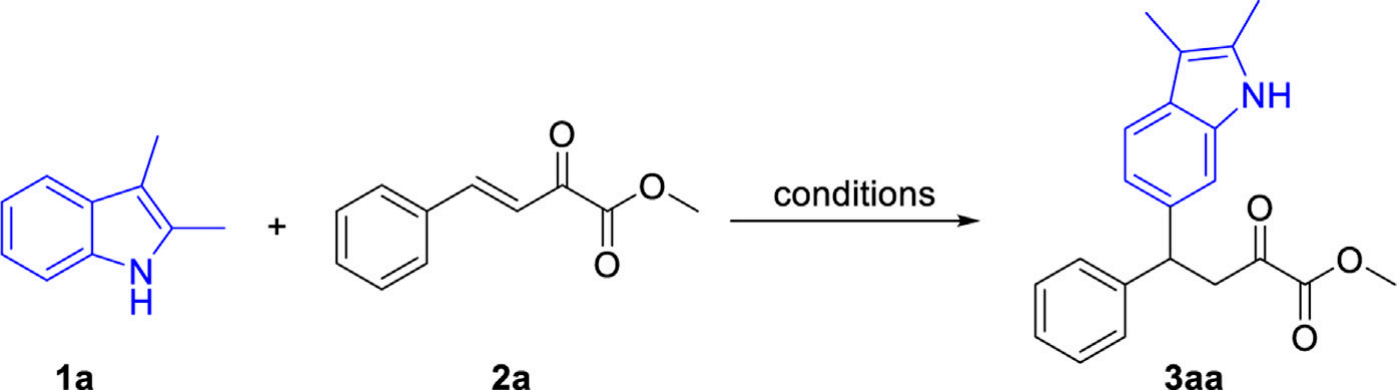					
**Entry**	**Catalyst**	**Sol**	**Temp. (°C)**	**1a:2a**	**Yield (%)^b^**
1	BSA	Toluene	30	1.2:1	42
2	PTS	Toluene	30	1.2:1	54
3	diphenyl phosphite	Toluene	30	1.2:1	13
4	PPTS	Toluene	30	1.2:1	47
5	4-Cl-BSA	Toluene	30	1.2:1	37
6	FeCl_3_	Toluene	30	1.2:1	40
7	Sc(OTf)_3_	Toluene	30	1.2:1	41
8	PTS	DCM	30	1.2:1	30
9	PTS	DCE	30	1.2:1	39
10	PTS	THF	30	1.2:1	trace
11	PTS	DMSO	30	1.2:1	10
12	PTS	MeCN	30	1.2:1	85
13	PTS	MeOH	30	1.2:1	16
14	PTS	MeCN	0	1.2:1	69
15	PTS	MeCN	40	1.2:1	65
16	PTS	MeCN	50	1.2:1	33
17	PTS	MeCN	30	1:1.2	74
**18**	**PTS**	**MeCN**	**30**	**1:1.5**	**92**
19	PTS	MeCN	30	1:1	34
20	PTS	MeCN	30	1.5:1	83

^a^Reaction conditions: **1a** (0.2 mmol), **2a**, Catalyst (20 mol%), solvent (2 ml), 24 h, ^b^Isolated yields. BSA: benzenesulfonic acid. PTS: *p*-toluenesulfonic acid. PPTS: pyridinium *p*-toluenesulfonate. 4-Cl-BSA: 4-chlorobenzenesulfonic acid.

The optimized reaction conditions.

**SCHEME 3:**
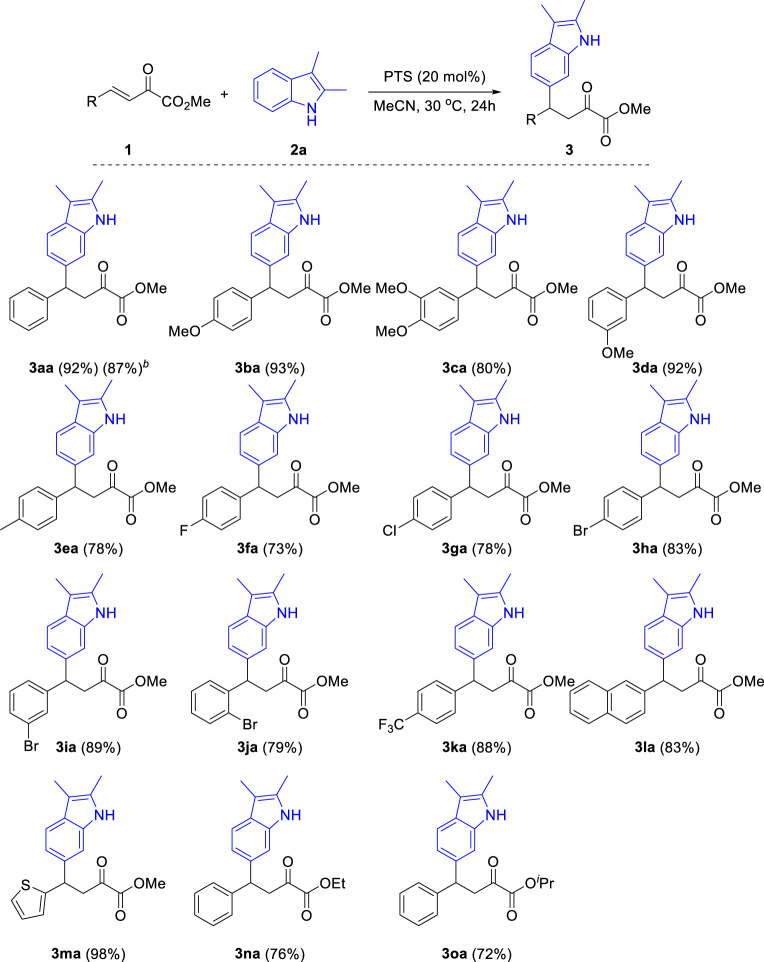
Substrate scope of β,γ-unsaturated α-ketoesters. ^a^Reaction conditions: β,γ-unsaturated α-ketoester **1** (0.2 mmol), Indoles **2a** (0.3 mmol), PTS (20 mol%), 30°C, 24 h, isolated yields. ^b^ **1a** (3 mmol), Indoles **2a** (4.5 mmol).

With the optimal reaction conditions in hand, we turned our attention to examining the generality of that reaction. Firstly, as shown in Scheme 3, under optimal reaction conditions, various substituted *β,γ*-unsaturated *α*-ketoesters reacted with 2,3-dimethyl indole **2a** and produced the corresponding products in good to excellent yields. Both electron-donating and electron-withdrawing group substituted *β,γ*-unsaturated *α*-ketoesters were tolerated in this reaction. The electronic properties of the substituents affect the efficiency of this reaction. Generally, *β,γ*-unsaturated *α*-ketoesters with electron-donating groups (**3ba-3ea**) gave higher yields than that with electron-withdrawing groups (**3fa-3ka**). The steric effect of the substituents has little influence to the yield of this reaction (**3ba** *vs*. **3da**, **3ha** *vs*. **3ja**). Notably, halides including fluoro-, chloro- and bromo-groups were compatible in this reaction. Furthermore, heteroarenes such as 2-naphthyl (**3la**) and thienyl (**3ma**) substituted enoneates were also compatible in this reaction and produced the corresponding products in 83% and 98% yields, respectively. In addition, the reaction also gave good yields when ethyl and isopropyl esters were used (**3na**, **3oa**).

**SCHEME 4:**
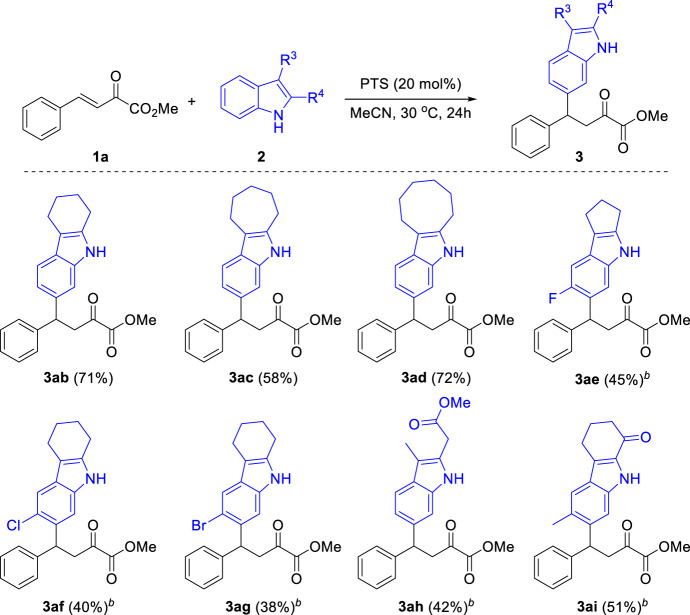
Substrate Scope of Indoles. ^a^Reaction conditions: β,γ-unsaturated α-ketoester **1a** (0.2 mmol), Indoles **2** (0.3 mmol), PTS (20 mol%), 30°C, 24 h, isolated yields. ^b^60°C.

Then, we examined the scope of indoles for this reaction. As shown in Scheme 4, a range of 2,3-disubstituted indoles were successfully applied in this reaction, providing the corresponding C6 functionalized products in moderate to good yields. When tetrahydro-1*H*-carbazole was subjected to the optimized conditions, the desired product **3 ab** was obtained in 71% yield. Other analogues with six-, seven- and five-membered fused rings were also tolerated in this reaction **(3ac-3ae)**. Besides, several functional groups including halides **(3ae-3ag)**, ester **(3ah)** and ketone **(3ai)** were compatible in this reaction. It should be noted that the substitution at both C2 and C3 positions of indole were essential for this reaction. When 2-methyl-1*H*-indole was used in this reaction, a mixture of isomers were obtained, while 3-methyl-1*H*-indole led to the formation of 9*H*-pyrrolo[1,2-*a*]indole (Sun et al., 2016). Other heterocycles such as benzofuran, benzothiophene, carbazole and quinoline were failed in this reaction.

**SCHEME 5:**
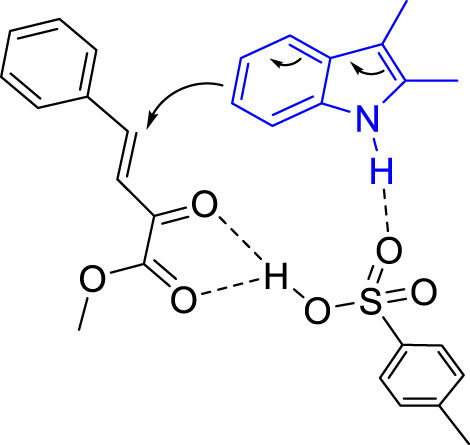
Proposed Reaction Modela.

On the basis of these results and the previous literatures, we proposed a plausible reaction model to illustrate the regioselectivity of this reaction. The Brønsted acid served as a bifunctional catalyst to activate both the indole and the *β,γ*-unsaturated *α*-ketoesters (Scheme 5).

In summary, we have developed a metal-free catalytic approach for the remote C6-functionalization of 2,3-disubstituted indoles. In the presence of catalytic amounts of Brønsted acid, the *β,γ*-unsaturated *α*-ketoesters react with 2,3-disubstituted indoles at the C6 position selectively. Under mild reaction conditions, a range of C6-functionalized indoles were prepared with good yields and excellent regioselectivity. This methodology provides a concise and efficient route for the synthesis of C6-functionalized indole derivatives.

## Data Availability

The original contributions presented in the study are included in the article/Supplementary Materials, further inquiries can be directed to the corresponding author.
