# Supplementary material for: Brønsted acid catalyzed remote C6 functionalization of 2,3-disubstituted indoles with β,γ-unsaturated α-ketoester
Source: Front Chem. 2022 Sep 13;10:992398. doi: 10.3389/fchem.2022.992398 (PMC9513241; doi:10.3389/fchem.2022.992398)
Supplement: Supplementary file 1 [file DataSheet1.docx]

**Supporting Information**

Brønsted Acid Catalyzed Remote C6 Functionalization of 2,3-Disubstituted Indoles with *β,γ*-unsaturated *α*-ketoester

You-Ya Zhang^1^, Lin Li^1^, Xiang-Zhi Zhang^1^, Jin-Bao Peng^1,^*

^1^School of Biotechnology and Health Sciences, Wuyi University, Jiangmen, Guangdong 529020, P. R. China; orcid.org/0000-0002-0568-7740; E-mail: pengjb_05@126.com

**Table of Contents**

[**1. General Information 1**](#_Toc16862)

[**2 Preparation of the Compounds 1a-1o 2**](#_Toc8377)

[**3 Preparation of the Compound 2h 3**](#_Toc8377)

[**4 Optimization of Reaction Conditions 3**](#_Toc10167)

[**5 General Procedure 5**](#_Toc23293)

[**6 Experimental Characterization Data for the Starting 5**](#_Toc3995)

[**Materials 5**](#_Toc20704)

[**7 Experimental Characterization Data for the Products 8**](#_Toc20577)

[**8 References**](#_Toc3970) **19**

[**9 Copies of NMR Spectra for Compounds 2**](#_Toc8613)**0**

# 1. General Information

**Reagents, solvents and analytical methods:**

Unless otherwise noted, all reactions were carried out under a nitrogen atmosphere. All reagents were from commercial sources and used as received without further purification. All solvents were dried by standard techniques and distilled prior to use. Column chromatography was performed on silica gel (200-300 meshes) using petroleum ether (bp. 60~90 °C) and ethyl acetate as eluent. ^1^NMR spectra were recorded on a Bruker Avance operating at for ^1^H NMR at 500 MHz, ^13^C NMR at 126 MHz and ^19^F NMR at 471 MHz and spectral data were reported in ppm relative to tetramethylsilane (TMS) as internal standard and CDCl_3_ (^1^H NMR δ 7.27, ^13^C NMR δ 77.0) as solvent. High-resolution mass spectra (HRMS) is produced by Thermo Fisher Scientific. Its main body is composed of two parts: Thermo Scientific's UltiMate 3000 Series liquid system and Thermo Scientific Q-Exactive combined quadrupole Orbitrap mass spectrometer. All coupling constants (*J*) are reported in Hz. The following abbreviations were used to describe peak splitting patterns when appropriate: s = singlet, d = doublet, dd = double doublet, ddd = double doublet of doublets, t = triplet, dt = double triplet, q = quatriplet, m = multiplet, br = broad.

# 2 Preparation of the Compounds 1a-1o

Compounds **1** were prepared according to the previous literature.^S1^

**Figure S1** Substrates of β,γ-unsaturated α-ketoester

To a solution of corresponding aldehyde (50 mmol) pyruvic acid (3.5 ml 50mmol) in methanol (8 mL), and the mixture was cooled to 0 °C. A solution of KOH (4.21 g, 75 mmol) in methanol (19 mL) was added dropwise at 0 °C. The first 1 equiv of the base solution were added dropwise. Then the reaction was warmed to room temperature and the rest of the base solution was added quickly. The reaction mixture was stirred at room temperature for 1h and then 0 °C overnight, the precipitate filtered and washed with chilled methanol and Et_2_O to affording the corresponding potassium salt as a solid.

To the corresponding alcohol (20 mL) at 0 °C, acetyl chloride (10 mL) was added dropwise until generation of HCl ceased. Then, the previously prepared potassium salt (24 mmol) was added at 0 °C, the reaction mixture was warmed to room temperature, stirred for 2h and then refluxed overnight. The solvent was removed by evaporation and water (20 mL) was added to the crude product, which was then extracted with dichloromethane (20 mL x 2). The combined organic phase was washed with a saturated solution of NaHCO_3_ (15 mL x 2) and then with water (20 mL). The combined organic fractions were dried with anhydrous Na2SO4, and distilled of at reduced pressure. The residue was purified by column chromatography (Hexane/AcOEt) to give the corresponding *α,β*-unsaturated ketoesters.

# 3 Preparation of the Compounds 2h

Compounds **2h** were prepared according to the previous literature.^S2^

**Figure S2** Substrates of Indol

# 4 Optimization of Reaction Conditions

**Table S1. Optimization of the Catalyst.^[a]^**

| Entry | Catalyst | Yield (%)^[b]^ |
| --- | --- | --- |
| 1 | benzenesulfonic acid | 42 |
| 2 | PTS | 54 |
| 3 | DIPHENYL PHOSPHITE | 13 |
| 4 | (1R)-(-)-10-Camphorsulfonic acid | trace |
| 5 | PPTS | 47 |
| 6 | 4-Chlorobenzenesulfonic acid | 37 |
| 7 | FeCl_3_ | 40 |
| 8 | Sc(OTf)_3_ | 41 |
| 9 | 1,1'-Binaphthyl-2,2'-diyl hydrogenphosphate | trace |
| 10 | CH_3_COOH | trace |
| [a] Reaction conditions: **1a** (0.2 mmol), **2a** (0.24 mmol), Catalyst (20 mol%), Toluene (1.5 mL), N_2_ atmosphere, 30 ^o^C for 24 h. [b] Isolated yield. | | |

**Table S2. Optimization of Solvent.^[a]^**

| Entry | Solvent | Yield (%)^[b]^ |
| --- | --- | --- |
| 1 | DCM | 30 |
| 2 | DCE | 39 |
| 3 | THF | trace |
| 4 | DMSO | 10 |
| 5 | DMF | trace |
| 6 | 1,4-Dioxane | trace |
| 7 | MeCN | 85 |
| 8 | MeOH | 16 |
| [a] Reaction conditions: **1a** (0.2 mmol), **2a** (0.24 mmol), PTS (20 mol%),Solvent (1.5 mL), N_2_ atmosphere,30 ^o^C for 24 h. [b] Isolated yield. | | |

**Table S3. Optimization of Temperature.^[a]^**

| Entry | Temp. | Yield (%)^[b]^ |
| --- | --- | --- |
| 1 | 0 | 69 |
| 2 | 30 | 85 |
| 3 | 40 | 65 |
| 4 | 50 | 33 |
| [a] Reaction conditions: **1a** (0.2 mmol), **2a** (0.24 mmol), PTS (20 mol%), MeCN (1.5 mL), N_2_ atmosphere, T ^o^C for 24 h. [b] Isolated yield. | | |

**Table S4. Optimization of the proportion of raw materials.^[a]^**

| Entry | 1a:2a | Yield (%)^[b]^ |
| --- | --- | --- |
| 1 | 1.2:1 | 85 |
| 2 | 1:1.2 | 74 |
| 3 | 1:1.5 | 92 |
| 4 | 1:1 | 34 |
| 6 | 1.5:1 | 83 |
| [a] Reaction conditions: **1a** (0.2 mmol), **2a** (x mmol), PTS (20 mol%),MeCN (1.5 mL),N_2_ atmosphere, 30 ^o^C for 24 h. [b] Isolated yield. | | |

# 5 General Procedure

**1** (0.2 mmol, 1 equiv), **2** (0.3 mmol,1.5 equiv), PTS (20 mol%) were transferred into a 15 mL tube. Then the tube was sealed with a septum. The tube was connected to an nitrogen-vacuum line, evacuated and backfilled with N_2_ (x3). MeCN (1.5 mL) was added to the reaction tube. The reaction mixture was stirred at room temperature for 24 hours. The mixture was concentrated under reduced pressure and the residue was purified by flash chromatography on silica gel eluting with petroleum ether/EtOAc (v/v = 10:1 to 4:1) to afford the products **3**.

# 6 Experimental Characterization Data for the Starting

# Materials

**Methyl (E)-2-oxo-4-phenylbut-3-enoate (1a)** (Known compound).^S1^ The compound was prepared according to the general procedure to give a yellow solid (56% yield).

**Methyl (E)-4-(4-methoxyphenyl)-2-oxobut-3-enoate (1b)** (Known compound).^S1^ The compound was prepared according to the general procedure to give a yellow solid (45% yield).

**Methyl (E)-4-(3,4-dimethoxyphenyl)-2-oxobut-3-enoate (1c)** (Known compound).^S1^ The compound was prepared according to the general procedure to give a yellow solid (32% yield).

**Methyl (E)-4-(3-methoxyphenyl)-2-oxobut-3-enoate (1d)** (Known compound).^S1^ The compound was prepared according to the general procedure to give a yellow solid (52% yield).

**Methyl (E)-2-oxo-4-(p-tolyl)but-3-enoate (1e)** (Known compound).^S1^ The compound was prepared according to the general procedure to give a yellow solid (55% yield).

**Methyl (E)-4-(4-fluorophenyl)-2-oxobut-3-enoate (1f)** (Known compound).^S1^ The compound was prepared according to the general procedure to give a yellow solid (40% yield).

**Methyl (E)-4-(4-chlorophenyl)-2-oxobut-3-enoate (1g)** (Known compound).^S1^ The compound was prepared according to the general procedure to give a yellow solid (39% yield).

**Methyl (E)-4-(4-bromophenyl)-2-oxobut-3-enoate (1h)** (Known compound).^S1^ The compound was prepared according to the general procedure to give a yellow solid (42% yield).

**Methyl (E)-4-(3-bromophenyl)-2-oxobut-3-enoate (1i)** (Known compound).^S1^ The compound was prepared according to the general procedure to give a yellow solid (35% yield).

**Methyl (E)-4-(2-bromophenyl)-2-oxobut-3-enoate(1j)** (Known compound).^S1^ The compound was prepared according to the general procedure to give a yellow solid (48% yield).

**Methyl (E)-2-oxo-4-(4-(trifluoromethyl)phenyl)but-3-enoate (1k)** (Known compound).^S1^ The compound was prepared according to the general procedure to give a yellow solid (53% yield).

**Methyl (E)-4-(naphthalen-2-yl)-2-oxobut-3-enoate(1l)** (Known compound).^S1^ The compound was prepared according to the general procedure to give a yellow solid (45% yield).

**Methyl (E)-2-oxo-4-(thiophen-2-yl)but-3-enoate (1m)** (Known compound).^S1^ The compound was prepared according to the general procedure to give a yellow solid (47% yield).

**Ethyl (E)-2-oxo-4-phenylbut-3-enoate (1n)** (Known compound).^S1^ The compound was prepared according to the general procedure to give a yellow oil (35% yield).

**Isopropyl (E)-2-oxo-4-phenylbut-3-enoate (1o)** (Known compound).^S1^ The compound was prepared according to the general procedure to give a yellow oil (32% yield).

**Methyl 2-(3-methyl-1H-indol-2-yl)acetate (2h)** (Known compound).^S2^ The compound was prepared according to the general procedure to give a yellow solid (48% yield).

# 7 Experimental Characterization Data for the Products

**Methyl 4-(2,3-dimethyl-1H-indol-6-yl)-2-oxo-4-phenylbutanoate (3aa)**

From methyl (E)-2-oxo-4-phenylbut-3-enoate (38 mg, 0.2 mmol) and 2,3-dimethyl-1H-indole (43.6mg, 0.3 mmol), following the general procedure, the title compound (61.6mg, 92%) was obtained as a yellow oil. **R*_f_*** = 0.5 (petroleum ether / ethyl acetate = 5:1).

**^1^H NMR (500 MHz, CDCl3)** δ 7.61 (s, 1H), 7.34 (d, *J* = 8.1 Hz, 1H), 7.24 (dd, *J* = 6.2, 3.2 Hz, 4H), 7.15 (ddd, *J* = 8.5, 5.7, 2.7 Hz, 1H), 7.07 (s, 1H), 6.95 (dd, *J* = 8.1, 1.3 Hz, 1H), 4.73 (s, 1H), 3.76 (s, 3H), 3.66 (m, 2H), 2.28 (d, *J* = 5.0 Hz, 3H), 2.16 (s, 3H).

**^13^C NMR (126 MHz, CDCl3)** δ 192.7, 161.4, 144.1, 136.1, 135.4, 131.0, 128.6, 128.3, 127.8, 126.5, 119.3, 118.2, 109.4, 107.0, 53.0, 45.8, 45.7, 11.6, 8.5.

**HRMS (ESI) m/z**: [M+Na]^+^ Calcd for C_21_H_21_NO_3_Na^+^ 358.1414; Found 358.1415.

**Methyl 4-(2,3-dimethyl-1H-indol-6-yl)-4-(4-methoxyphenyl)-2-oxobutanoate (3ba)**

From methyl (E)-4-(4-methoxyphenyl)-2-oxobut-3-enoate (44 mg, 0.2 mmol) and 2,3-dimethyl-1H-indole (43.6mg, 0.3 mmol), following the general procedure, the title compound (67.9mg, 93%) was obtained as a yellow oil. **R*_f_*** = 0.3 (petroleum ether / ethyl acetate = 5:1).

**^1^H NMR (500 MHz, CDCl3)** δ 7.61 (s, 1H), 7.37 – 7.29 (m, 1H), 7.17 (m, 2H), 7.11 – 7.04 (m, 1H), 6.93 (dd, *J* = 8.1, 1.2 Hz, 1H), 6.78 (d, *J* = 8.7 Hz, 2H), 4.68 (t, *J* = 7.7 Hz, 1H), 3.87 – 3.71 (m, 6H), 3.71 – 3.53 (m, 2H), 2.28 (d, *J* = 5.2 Hz, 3H), 2.17 (d, *J* = 11.0 Hz, 3H).

**^13^C NMR (126 MHz, CDCl3)** δ 192.8, 161.5, 158.1, 136.5, 136.3, 135.4, 130.9, 128.8, 128.2, 119.2, 118.2, 114.0, 109.3, 107.0, 55.3, 53.1, 45.9, 45.0, 11.6, 8.5.

**HRMS (ESI) m/z**: [M+Na]^+^ Calcd for C_22_H_23_NO_4_Na^+^ 388.1519; Found 388.1524.

**Methyl 4-(3,4-dimethoxyphenyl)-4-(2,3-dimethyl-1H-indol-6-yl)-2-oxobutanoate (3ca)**

From methyl (E)-4-(3,4-dimethoxyphenyl)-2-oxobut-3-enoate (56.1 mg, 0.2 mmol) and 2,3-dimethyl-1H-indole (43.6mg, 0.3 mmol), following the general procedure, the title compound (63.2mg, 80%) was obtained as a yellow oil. **R*_f_*** = 0.2 (petroleum ether / ethyl acetate = 5:1).

**^1^H NMR (500 MHz, CDCl3)** δ 7.72 (s, 1H), 7.35 (d, *J* = 8.1 Hz, 1H), 7.07 (s, 1H), 6.95 (dd, *J* = 8.2, 1.4 Hz, 1H), 6.81 – 6.75 (m, 3H), 4.69 (t, *J* = 7.7 Hz, 1H), 3.81 (s, 3H), 3.79 (s, 3H), 3.77 (s, 3H), 3.63 (dd, *J* = 7.7, 2.9 Hz, 2H), 2.29 (s, 3H), 2.17 (s, 3H).

**^13^C NMR (126 MHz, CDCl3)** δ 192.8, 161.5, 148.9, 147.6, 136.7, 136.3, 135.4, 131.0, 128.2, 119.5, 119.1, 118.1, 111.5, 111.2, 109.3, 106.9, 55.9, 55.9, 53.0, 45.9, 45.5, 11.6, 8.5.

**HRMS (ESI) m/z**: [M+Na]^+^ Calcd for C_23_H_25_NO_5_ Na^+^ 418.1731; Found 418.1738.

**Methyl 4-(2,3-dimethyl-1H-indol-6-yl)-4-(3-methoxyphenyl)-2-oxobutanoate (3da)**

From methyl (E)-4-(3-methoxyphenyl)-2-oxobut-3-enoate (44 mg, 0.2 mmol) and 2,3-dimethyl-1H-indole (43.6mg, 0.3 mmol), following the general procedure, the title compound (67.2mg, 92%) was obtained as a yellow oil. **R*_f_*** = 0.4 (petroleum ether / ethyl acetate = 5:1).

**^1^H NMR (500 MHz, CDCl3)** δ 7.67 (s, 1H), 7.33 (d, *J* = 8.1 Hz, 1H), 7.16 (t, *J* = 7.9 Hz, 1H), 7.07 (s, 1H), 6.95 (dd, *J* = 8.1, 1.3 Hz, 1H), 6.86 – 6.80 (m, 2H), 6.69 (dd, *J* = 8.2, 2.1 Hz, 1H), 4.70 (t, *J* = 7.6 Hz, 1H), 3.75 (s, 3H), 3.72 (s, 3H), 3.66 – 3.62 (m, 2H), 2.27 (s, 3H), 2.15 (s, 3H).

**^13^C NMR (126 MHz, CDCl3)** δ 192.6, 161.4, 159.7, 145.7, 135.9, 135.4, 131.0, 129.6, 128.3, 120.2, 119.1, 118.1, 113.9, 111.5, 109.4, 106.9, 55.2, 53.0, 45.7, 45.6, 11.6, 8.5.

**HRMS (ESI) m/z**: [M+H]^+^ Calcd for C_22_H_23_NO_4_H^+^ 366.1700; Found 366.1695.

**Methyl 4-(2,3-dimethyl-1H-indol-6-yl)-2-oxo-4-(p-tolyl)butanoate (3ea)**

From methyl (E)-2-oxo-4-(p-tolyl)but-3-enoate (40.9 mg, 0.2 mmol) and 2,3-dimethyl-1H-indole (43.6mg, 0.3 mmol), following the general procedure, the title compound (54.5mg, 78%) was obtained as a yellow oil. **R*_f_*** = 0.5 (petroleum ether / ethyl acetate = 5:1).

**^1^H NMR (500 MHz, CDCl3)** δ 7.58 (s, 1H), 7.33 (d, *J* = 8.1 Hz, 1H), 7.16 – 7.11 (m, 2H), 7.09 – 7.01 (m, 3H), 6.93 (dd, *J* = 8.1, 1.4 Hz, 1H), 4.70 (t, *J* = 7.7 Hz, 1H), 3.74 (s, 3H), 3.69 – 3.57 (m, 2H), 2.26 (d, *J* = 2.8 Hz, 6H), 2.15 (s, 3H).

**^13^C NMR (126 MHz, CDCl3)** δ 192.8, 161.4, 141.1, 136.3, 136.0, 135.4, 130.9, 129.3, 128.2, 127.7, 119.2, 118.1, 109.4, 106.8, 53.0, 45.8, 45.3, 21.0, 11.5, 8.5.

**HRMS (ESI) m/z**: [M+H]^+^ Calcd for C_22_H_23_NO_3_H^+^ 350.1751; Found 350.1747.

**Methyl 4-(2,3-dimethyl-1H-indol-6-yl)-4-(4-fluorophenyl)-2-oxobutanoate (3fa)**

From methyl (E)-4-(4-fluorophenyl)-2-oxobut-3-enoate (41.6 mg, 0.2 mmol) and 2,3-dimethyl-1H-indole (43.6mg, 0.3 mmol), following the general procedure, the title compound (51.6mg, 73%) was obtained as a yellow oil. **R*_f_*** = 0.5 (petroleum ether / ethyl acetate = 5:1).

**^1^H NMR (500 MHz, CDCl3)** δ 7.65 (s, 1H), 7.39 (d, *J* = 8.1 Hz, 1H), 7.24 (dd, *J* = 8.6, 5.4 Hz, 2H), 7.11 (s, 1H), 6.96 (t, *J* = 8.7 Hz, 3H), 4.75 (t, *J* = 7.5 Hz, 1H), 3.81 (s, 3H), 3.67 (dd, *J* = 15.3, 7.7 Hz, 2H), 2.35 (s, 3H), 2.20 (s, 3H).

**^13^C NMR (126 MHz, CDCl3)** δ 192.5, 161.4, 139.9 (d, *J* *_(C-F)_* = 3.0 Hz), 135.9, 135.4, 131.2, 129.3 (d, *J* *_(C-F)_*= 8.0 Hz), 128.4, 119.1, 118.3, 115.4(d, *J* *_(C-F)_* = 21.4 Hz), 110.4, 109.3, 107.1, 53.1, 45.8, 45.0, 11.6, 8.5.

**^19^F NMR (471 MHz, CDCl3)** δ -116.80 (s).

**HRMS (ESI) m/z**: [M+H]^+^ Calcd for C_21_H_20_FNO_3_H^+^ 354.1500; Found 354.1494.

**Methyl 4-(4-chlorophenyl)-4-(2,3-dimethyl-1H-indol-6-yl)-2-oxobutanoate(3ga)**

From methyl (E)-4-(4-chlorophenyl)-2-oxobut-3-enoate (44.9 mg, 0.2 mmol) and 2,3-dimethyl-1H-indole (43.6mg, 0.3 mmol), following the general procedure, the title compound (57.6mg,78%) was obtained as a yellow oil. **R*_f_*** = 0.5 (petroleum ether / ethyl acetate = 5:1).

**^1^H NMR (500 MHz, CDCl3)** δ 7.74 (s, 1H), 7.43 (d, *J* = 8.1 Hz, 1H), 7.28 – 7.21 (m, 4H), 7.10 (s, 1H), 6.98 (d, *J* = 8.0 Hz, 1H), 4.76 (t, *J* = 7.5 Hz, 1H), 3.83 (s, 3H), 3.75 – 3.64 (m, 2H), 2.32 (s, 3H), 2.24 (s, 3H).

**^13^C NMR (126 MHz, CDCl3)** δ 192.4, 161.3, 142.7, 135.4, 135.3, 132.1, 131.2, 129.2, 128.6, 128.3, 119.0, 118.2, 109.3, 106.9, 53.1, 45.5, 45.0, 11.5, 8.5.

**HRMS (ESI) m/z**: [M+Na]^+^ Calcd for C_21_H_20_ClNO_3_Na^+^ 392.1024; Found 392.1026.

**Methyl 4-(4-bromophenyl)-4-(2,3-dimethyl-1H-indol-6-yl)-2-oxobutanoate (3ha)**

From methyl (E)-4-(4-bromophenyl)-2-oxobut-3-enoate (53.9 mg, 0.2 mmol) and 2,3-dimethyl-1H-indole (43.6mg, 0.3 mmol), following the general procedure, the title compound (68.6mg, 83%) was obtained as a yellow oil. **R*_f_*** = 0.5 (petroleum ether / ethyl acetate = 5:1).

**^1^H NMR (500 MHz, CDCl3)** δ 7.71 (s, 1H), 7.39 (d, *J* = 8.4 Hz, 3H), 7.19 – 7.12 (m, 2H), 7.09 (s, 1H), 6.94 (dd, *J* = 8.2, 1.3 Hz, 1H), 4.73 (t, *J* = 7.6 Hz, 1H), 3.82 (s, 3H), 3.66 (m, 2H), 2.33 (s, 3H), 2.21 (s, 3H).

**^13^C NMR (126 MHz, CDCl3)** δ 192.3, 161.3, 143.2, 135.4, 135.3, 131.6, 131.2, 129.6, 128.4, 120.3, 119.0, 118.2, 109.3, 107.0, 53.1, 45.4, 45.1, 11.5, 8.5.

**HRMS (ESI) m/z**: [M+Na]^+^ Calcd for C_21_H_20_BrNO_3_Na^+^ 436.0519; Found 436.0518.

**Methyl 4-(3-bromophenyl)-4-(2,3-dimethyl-1H-indol-6-yl)-2-oxobutanoate (3ia)**

From methyl (E)-4-(3-bromophenyl)-2-oxobut-3-enoate (53.9 mg, 0.2 mmol) and 2,3-dimethyl-1H-indole (43.6mg, 0.3 mmol), following the general procedure, the title compound (73.5mg, 89%) was obtained as a yellow oil.**R*_f_*** = 0.5 (petroleum ether / ethyl acetate = 5:1).

**^1^H NMR (500 MHz, CDCl3)** δ 7.66 (s, 1H), 7.40 (s, 1H), 7.36 (d, *J* = 8.1 Hz, 1H), 7.28 (d, *J* = 7.9 Hz, 1H), 7.19 (d, *J* = 7.8 Hz, 1H), 7.10 (m, 2H), 6.92 (dd, *J* = 8.1, 1.1 Hz, 1H), 4.69 (t, *J* = 7.6 Hz, 1H), 3.78 (s, 3H), 3.69 – 3.57 (m, 2H), 2.30 (s, 3H), 2.17 (s, 3H).

**^13^C NMR (126 MHz, CDCl3)** δ 192.2, 161.3, 146.6, 135.4, 135.2, 131.2, 130.9, 130.2, 129.6, 128.5, 126.5, 122.7, 119.1, 118.4, 109.4, 107.1, 53.2, 45.4, 45.4, 11.6, 8.5.

**HRMS (ESI) m/z**: [M+H]^+^ Calcd for C_21_H_20_BrNO_3_H^+^ 414.0699; Found 414.0693.

**Methyl 4-(2-bromophenyl)-4-(2,3-dimethyl-1H-indol-6-yl)-2-oxobutanoate (3ja)**

From methyl (E)-4-(2-bromophenyl)-2-oxobut-3-enoate (53.9 mg, 0.2 mmol) and 2,3-dimethyl-1H-indole (43.6mg, 0.3 mmol), following the general procedure, the title compound (65.3mg, 79%) was obtained as a yellow oil. **R*_f_*** = 0.5 (petroleum ether / ethyl acetate = 5:1).

**^1^H NMR (500 MHz, CDCl3)** δ 7.63 (s, 1H), 7.54 – 7.51 (m, 1H), 7.35 (d, *J* = 8.1 Hz, 1H), 7.24 (dd, *J* = 4.8, 2.9 Hz, 1H), 7.22 – 7.18 (m, 1H), 7.11 (s, 1H), 7.02 (td, *J* = 7.7, 1.8 Hz, 1H), 6.97 (dd, *J* = 8.2, 1.4 Hz, 1H), 5.23 (dd, *J* = 8.3, 6.9 Hz, 1H), 3.78 (s, 3H), 3.73 (dd, *J* = 17.6, 8.6 Hz, 1H), 3.53 (dd, *J* = 17.6, 6.7 Hz, 1H), 2.28 (s, 3H), 2.16 (s, 3H).

**^13^C NMR (126 MHz, CDCl3)** δ 192.1, 161.4, 143.0, 135.3, 134.3, 133.4, 131.1, 128.9, 128.4, 128.1, 127.7, 124.9, 119.4, 118.2, 109.9, 107.0, 53.1, 45.3, 44.5, 11.6, 8.5

**HRMS (ESI) m/z**: [M+H]^+^ Calcd for C_21_H_20_BrNO_3_H^+^ 414.0699; Found 414.0695.

**Methyl 4-(2,3-dimethyl-1H-indol-6-yl)-2-oxo-4-(4-(trifluoromethyl)phenyl)butanoate (3ka)**

From methyl (E)-2-oxo-4-(4-(trifluoromethyl)phenyl)but-3-enoate (51.6 mg, 0.2 mmol) and 2,3-dimethyl-1H-indole (43.6mg, 0.3 mmol), following the general procedure, the title compound (70.9mg, 88%) was obtained as a yellow oil. **R*_f_*** = 0.4 (petroleum ether / ethyl acetate = 5:1).

**^1^H NMR (500 MHz, CDCl3)** δ 7.69 (s, 1H), 7.48 (d, *J* = 8.2 Hz, 2H), 7.36 (dd, *J* = 8.1, 2.2 Hz, 3H), 7.06 (d, *J* = 0.8 Hz, 1H), 6.91 (dd, *J* = 8.1, 1.5 Hz, 1H), 4.77 (t, *J* = 7.5 Hz, 1H), 3.78 (s, 3H), 3.73 – 3.61 (m, 2H), 2.28 (s, 3H), 2.16 (s, 3H).

**^13^C NMR (126 MHz, CDCl_3_)** δ 192.2, 161.3, 148.2, 135.4, 135.0, 131.4, 128.6, 128.5, 128.2, 125.6 (q, *J* = 3.6 Hz), 119.1, 118.4, 109.4, 107.1, 53.2, 45.5, 45.3, 11.6, 8.5.

**^19^F NMR (471 MHz, CDCl3)** δ -62.43 (s).

**HRMS (ESI) m/z**: [M+Na]^+^ Calcd for C_22_H_20_F_3_NO_3_Na^+^ 428.1287; Found 428.1287.

**Methyl 4-(2,3-dimethyl-1H-indol-6-yl)-4-(naphthalen-2-yl)-2-oxobutanoate (3la)**

From methyl (E)-4-(naphthalen-2-yl)-2-oxobut-3-enoate (48.0 mg, 0.2 mmol) and 2,3-dimethyl-1H-indole (43.6mg, 0.3 mmol), following the general procedure, the title compound (63.9mg, 83%) was obtained as a yellow oil. **R*_f_*** = 0.5 (petroleum ether / ethyl acetate = 5:1).

**^1^H NMR (500 MHz, CDCl3)** δ 7.79 – 7.66 (m, 4H), 7.58 (s, 1H), 7.46 – 7.30 (m, 4H), 7.10 (s, 1H), 6.99 (dd, *J* = 8.2, 1.2 Hz, 1H), 5.01 – 4.79 (m, 1H), 3.88 – 3.66 (m, 5H), 2.27 (s, 3H), 2.15 (s, 3H).

**^13^C NMR (126 MHz, CDCl3)** δ 192.7, 161.5, 141.5, 135.9, 135.4, 133.5, 132.3, 131.1, 128.3, 127.9, 127.7, 126.9, 126.2, 125.7, 125.6, 119.4, 118.2, 109.6, 107.02, 53.1, 45.8, 45.6, 11.6, 8.5.

**HRMS (ESI) m/z**: [M+Na]^+^ Calcd for C_25_H_23_NO_3_Na^+^ 408.1570; Found 408.1578.

**Methyl 4-(2,3-dimethyl-1H-indol-6-yl)-2-oxo-4-(thiophen-2-yl)butanoate (3ma)**

From methyl (E)-2-oxo-4-(thiophen-2-yl)but-3-enoate (39.2 mg, 0.2 mmol) and 2,3-dimethyl-1H-indole (43.6mg, 0.3 mmol), following the general procedure, the title compound (66.8mg, 98%) was obtained as a yellow oil.**R*_f_*** = 0.5 (petroleum ether / ethyl acetate = 4:1).

**^1^H NMR (500 MHz, CDCl3)** δ 7.72 (s, 1H), 7.40 (d, *J* = 8.2 Hz, 1H), 7.16 (s, 1H), 7.13 (d, *J* = 5.1 Hz, 1H), 7.02 (d, *J* = 8.1 Hz, 1H), 6.91 – 6.89 (m, 1H), 6.87 (d, *J* = 3.4 Hz, 1H), 4.98 (m, 1H), 3.80 (d, *J* = 1.8 Hz, 3H), 3.71 (d, *J* = 7.5 Hz, 2H), 2.32 (s, 3H), 2.20 (s, 3H).

**^13^C NMR (126 MHz, CDCl3)** δ 192.0, 161.2, 148.6, 135.7, 135.3, 131.3, 128.6, 126.7, 124.1, 124.1, 118.9, 118.2, 109.4, 107.0, 53.1, 47.1, 41.5, 11.6, 8.5.

**HRMS (ESI) m/z**: [M+Na]^+^ Calcd for C_19_H_19_NO_3_SNa^+^ 364.0978; Found 364.0984.

**Ethyl 4-(2,3-dimethyl-1H-indol-6-yl)-2-oxo-4-phenylbutanoate(3na)**

From methyl ethyl (E)-2-oxo-4-phenylbut-3-enoate (40.8 mg, 0.2 mmol) and 2,3-dimethyl-1H-indole (43.6mg, 0.3 mmol), following the general procedure, the title compound (53.1mg, 76%) was obtained as a yellow oil. **R*_f_*** = 0.5 (petroleum ether / ethyl acetate = 5:1).

**^1^H NMR (500 MHz, CDCl3)** δ 7.66 (s, 1H), 7.38 (d, *J* = 8.1 Hz, 1H), 7.29 (d, *J* = 3.3 Hz, 3H), 7.26 (d, *J* = 0.8 Hz, 1H), 7.20 – 7.17 (m, 1H), 7.13 (s, 1H), 6.99 (dd, *J* = 8.1, 1.5 Hz, 1H), 4.77 (t, *J* = 7.7 Hz, 1H), 4.25 (dt, *J* = 9.5, 6.0 Hz, 2H), 3.73 – 3.62 (m, 2H), 2.33 (s, 3H), 2.20 (s, 3H), 1.31 (d, *J* = 7.1 Hz, 3H).

**^13^C NMR (126 MHz, CDCl3)** δ 193.1, 161.1, 144.2, 136.1, 135.4, 131.0, 128.6, 128.3, 127.8, 126.4, 119.3, 118.1, 109.4, 106.9, 62.5, 45.8, 45.6, 14.0, 11.6, 8.5.

**HRMS (ESI) m/z**: [M+H]^+^ Calcd for C_22_H_23_NO_3_H^+^ 350.1751; Found 350.1746.

**Isopropyl 4-(2,3-dimethyl-1H-indol-6-yl)-2-oxo-4-phenylbutanoate (3oa)**

From isopropyl 4-(2,3-dimethyl-1H-indol-6-yl)-2-oxo-4-phenylbutanoate (43.6mg, 0.2 mmol) and 2,3-dimethyl-1H-indole (43.6mg, 0.3 mmol), following the general procedure, the title compound (52.3mg, 72%) was obtained as a yellow oil. **R*_f_*** = 0.5 (petroleum ether / ethyl acetate = 5:1).

**^1^H NMR (500 MHz, CDCl3)** δ 7.61 (s, 1H), 7.34 (d, *J* = 8.1 Hz, 1H), 7.27 – 7.24 (m, 4H), 7.15 (ddd, *J* = 8.5, 5.9, 2.4 Hz, 1H), 7.08 (s, 1H), 6.95 (dd, *J* = 8.1, 1.4 Hz, 1H), 5.03 (m, 1H), 4.73 (t, *J* = 7.7 Hz, 1H), 3.68 – 3.58 (m, 2H), 2.29 (s, 3H), 2.16 (s, 3H), 1.25 (t, *J* = 6.1 Hz, 6H).

**^13^C NMR (126 MHz, CDCl3)** δ 193.5, 160.7, 144.2, 136.2, 135.4, 131.0, 128.9, 128.6, 128.3, 127.9, 126.5, 119.3, 118.2, 109.5, 70.8, 45.9, 21.6, 21.6, 11.6, 8.5.

**HRMS (ESI) m/z**: [M+H]^+^ Calcd for C_23_H_25_NO_3_H^+^ 364.1907; Found 364.1902.

**Methyl 2-oxo-4-phenyl-4-(2,3,4,9-tetrahydro-1H-carbazol-7-yl)butanoate (3ab)**

From methyl (E)-2-oxo-4-phenylbut-3-enoate (38 mg, 0.2 mmol) and 2,3,4,9-tetrahydro-1H-carbazole (51.6 mg, 0.3 mmol), following the general procedure , the title compound (51.3mg, 71%) was obtained as a yellow oil. **R*_f_*** = 0.4 (petroleum ether / ethyl acetate = 5:1).

**^1^H NMR (500 MHz, CDCl3)** δ 7.65 (s, 1H), 7.39 (d, *J* = 8.1 Hz, 1H), 7.30 (dd, *J* = 5.6, 2.7 Hz, 4H), 7.20 (td, *J* = 5.7, 2.8 Hz, 1H), 7.15 (s, 1H), 7.00 (dd, *J* = 8.1, 1.3 Hz, 1H), 4.80 (dd, *J* = 9.5, 5.6 Hz, 1H), 3.81 (s, 3H), 3.76 – 3.65 (m, 2H), 2.69 (t, *J* = 5.7 Hz, 4H), 1.93 – 1.85 (m, 4H).

**^13^C NMR (126 MHz, CDCl3)** δ 192.7, 161.4, 144.2, 136.1, 135.9, 134.5, 128.6, 127.8, 126.6, 126.4, 119.3, 117.9, 110.0, 109.8, 67.1, 53.0, 45.7, 45.7, 23.3, 21.0.

**HRMS (ESI) m/z**: [M+H]^+^ Calcd for C_23_H_23_NO_3_H^+^362.1751; Found 362.1745.

**Methyl 4-(5,6,7,8,9,10-hexahydrocyclohepta[b]indol-3-yl)-2-oxo-4-phenylbutanoate (3ac)**

From methyl (E)-2-oxo-4-phenylbut-3-enoate (38 mg, 0.2 mmol) and 5,6,7,8,9,10-hexahydrocyclohepta[b]indole (55 mg, 0.3 mmol), following the general procedure , the title compound (43.5 mg, 58%) was obtained as a yellow oil. **R*_f_*** = 0.5 (petroleum ether / ethyl acetate = 5:1).

**^1^H NMR (500 MHz, CDCl3)**δ 7.62 (s, 1H), 7.36 (d, *J* = 8.2 Hz, 1H), 7.26 – 7.24 (m, 4H), 7.15 (ddd, *J* = 8.6, 5.7, 2.6 Hz, 1H), 7.10 (s, 1H), 6.95 (dd, *J* = 8.2, 1.4 Hz, 1H), 4.74 (t, *J* = 7.6 Hz, 1H), 3.77 (s, 3H), 3.66 (m, 2H), 2.79 – 2.73 (m, 4H), 1.86 (d, *J* = 5.0 Hz, 2H), 1.74 (dd, *J* = 6.9, 4.4 Hz, 4H)

**^13^C NMR (126 MHz, CDCl3)** δ 192.7, 161.5, 144.1, 137.8, 135.9, 134.4, 128.6, 128.2, 127.8, 126.5, 119.4, 118.0, 113.7, 109.5, 53.1, 45.7, 45.7, 31.9, 29.7, 28.8, 27.6, 24.8.

**HRMS (ESI) m/z**: [M+H]^+^ Calcd for C_24_H_25_NO_3_H^+^ 376.1907; Found 376.1901.

**Methyl 4-(6,7,8,9,10,11-hexahydro-5H-cycloocta[b]indol-3-yl)-2-oxo-4-phenylbutanoate (3ad)**

From methyl (E)-2-oxo-4-phenylbut-3-enoate (38 mg, 0.2 mmol) and 6,7,8,9,10,11-hexahydro-5H-cycloocta[b]indole (60 mg, 0.3 mmol), following the general procedure, the title compound (53.6 mg, 72%) was obtained as a yellow oil. **R*_f_*** = 0.5 (petroleum ether / ethyl acetate = 5:1).

**^1^H NMR (500 MHz, CDCl3)** δ 7.51 (s, 1H), 7.28 (d, *J* = 8.1 Hz, 1H), 7.20 – 7.15 (m, 4H), 7.09 – 7.05 (m, 1H), 7.00 (s, 1H), 6.87 (dd, *J* = 8.2, 1.3 Hz, 1H), 4.65 (t, *J* = 7.6 Hz, 1H), 3.67 (s, 3H), 3.63 – 3.56 (m, 2H), 2.73 – 2.66 (m, 4H), 1.61 (m, 4H), 1.37 – 1.29 (m, 4H).

**^13^C NMR (126 MHz, CDCl3)** δ 192.7, 161.4, 144.1, 135.8, 135.2, 128.6, 128.6, 127.9, 127.5, 126.5, 119.1, 117.9, 111.5, 109.6, 53.0, 45.8, 45.8, 45.8, 29.6, 29.5, 26.0, 25.9, 22.3.

**HRMS (ESI) m/z**: [M+H]^+^ Calcd for C_25_H_27_NO_3_H^+^390.2064; Found 390.2057.

**Methyl 4-(7-fluoro-1,2,3,4-tetrahydrocyclopenta[b]indol-6-yl)-2-oxo-4-phenylbutanoate (3ae)**

From methyl (E)-2-oxo-4-phenylbut-3-enoate (38 mg, 0.2 mmol) and 7-fluoro-1,2,3,4-tetrahydrocyclopenta[b]indole (53mg, 0.3 mmol), following the general procedure, the title compound (33.6 mg, 45%) was obtained as a yellow oil.**R*_f_*** = 0.4 (petroleum ether / ethyl acetate = 5:1).

**^1^H NMR (500 MHz, CDCl3)** δ 7.78 (s, 1H), 7.26 (dd, *J* = 6.4, 2.5 Hz, 4H), 7.17 (td, *J* = 5.9, 2.9 Hz, 1H), 7.03 (m, 2H), 4.98 (t, *J* = 7.6 Hz, 1H), 3.80 (s, 3H), 3.65 (m, 2H), 2.82 – 2.78 (m, 2H), 2.74 (t, *J* = 7.0 Hz, 2H), 2.52 – 2.46 (m, 2H).

**^13^C NMR (126 MHz, CDCl3)** δ 192.4, 161.4, 154.9, 145.9, 142.8 , 137.5, 128.6, 127.3( d, *J* =151.2 Hz), 123.8, 123.1 (d, *J* = 17.9 Hz), 119.9, 110.8 (d, *J* = 5.3 Hz), 104.3, 104.1, 53.1, 44.7, 39.6, 28.7, 26.0 , 24.4.

**^19^F NMR (471 MHz, CDCl3)** δ -127.70 (dd, *J* = 11.1, 6.3 Hz).

**HRMS (ESI) m/z**: [M+H]^+^ Calcd for C_22_H_20_FNO_3_H^+^ 366.1500; Found366.1496.

**Methyl 4-(6-chloro-2,3,4,9-tetrahydro-1H-carbazol-7-yl)-2-oxo-4-phenylbutanoate (3af)**

From methyl (E)-2-oxo-4-phenylbut-3-enoate (38 mg, 0.2 mmol) and 6-chloro-2,3,4,9-tetrahydro-1H-carbazole (62mg, 0.3 mmol), following the general procedure, the title compound (31.6 mg, 40%) was obtained as a yellow oil. **R*_f_*** = 0.4 (petroleum ether / ethyl acetate = 5:1).

**^1^H NMR (500 MHz, CDCl3)** δ 7.68 (s, 1H), 7.45 (s, 1H), 7.28 (d, *J* = 5.1 Hz, 4H), 7.20 (td, *J* = 8.8, 4.6 Hz, 1H), 7.08 (s, 1H), 5.29 (dd, *J* = 19.4, 11.8 Hz, 1H), 3.83 (s, 3H), 3.73 (m, 1H), 3.58 (m, 1H), 2.66 (m, 4H), 1.94 – 1.81 (m, 4H).

**^13^C NMR (126 MHz, CDCl3)** δ 192.3, 161.5, 142.6, 136.1, 134.6, 132.7, 128.6, 128.2, 127.8, 126.6, 125.0, 118.7, 110.4, 109.9, 53.1, 45.2, 42.1, 23.3, 23.2, 23.1, 20.9.

**HRMS (ESI) m/z**: [M+H]^+^ Calcd for C_23_H_22_ClNO_3_H^+^ 396.1361; Found 396.1367.

**Methyl 4-(6-bromo-2,3,4,9-tetrahydro-1H-carbazol-7-yl)-2-oxo-4-phenylbutanoate(3ag)**

From methyl (E)-2-oxo-4-phenylbut-3-enoate (38 mg, 0.2 mmol) and 6-bromo-2,3,4,9-tetrahydro-1H-carbazole (76 mg, 0.3 mmol), following the general procedure, the title compound (26.6 mg, 38%) was obtained as a yellow oil. **R*_f_*** = 0.5 (petroleum ether / ethyl acetate = 5:1).

**^1^H NMR (500 MHz, CDCl3)** δ 7.52 (s, 1H), 7.26 (d, *J* = 8.1 Hz, 1H), 7.17 – 7.15 (m, 3H), 7.07 (td, *J* = 5.7, 2.8 Hz, 1H), 7.01 (s, 1H), 6.86 (dd, *J* = 8.1, 1.3 Hz, 1H), 4.66 (t, *J* = 7.6 Hz, 1H), 3.68 (s, 3H), 3.61 – 3.53 (m, 2H), 2.55 (t, *J* = 5.7 Hz, 4H), 1.79 – 1.72 (m, 4H).

**^13^C NMR (126 MHz, CDCl3)** δ 192.7, 161.4, 144.1, 136.1, 135.9, 134.5, 128.6, 127.8, 126.6, 126.5, 119.3, 117.9, 110.0, 109.8, 53.0, 45.7, 45.7, 23.3, 23.3, 23.2, 21.0.

**HRMS (ESI) m/z**: [M+H]^+^ Calcd for C_23_H_22_BrNO_3_H^+^ 440.0856; Found 440.0850.

**Methyl 4-(2-(2-methoxy-2-oxoethyl)-3-methyl-1H-indol-6-yl)-2-oxo-4-phenylbutanoate (3ah)**

From methyl (E)-2-oxo-4-phenylbut-3-enoate (38 mg, 0.2 mmol) and methyl 2-(3-methyl-1H-indol-2-yl)acetate (60.9 mg, 0.3 mmol), following the general procedure, the title compound (33mg, 42%) was obtained as a yellow oil.**R*_f_*** = 0.3 (petroleum ether / ethyl acetate = 5:1).

**^1^H NMR (500 MHz, CDCl3)** δ 7.72 (d, *J* = 7.7 Hz, 1H), 7.34 (d, *J* = 8.2 Hz, 1H), 7.19 (d, *J* = 3.8 Hz, 5H), 7.09 (dd, *J* = 8.0, 3.1 Hz, 1H), 7.05 (s, 1H), 6.91 (d, *J* = 8.2 Hz, 1H), 4.67 (t, *J* = 7.8 Hz, 1H), 3.71 (d, *J* = 1.4 Hz, 2H), 3.62 – 3.58 (m, 2H), 3.57 (t, *J* = 3.0 Hz, 3H), 2.31 (d, *J* = 4.5 Hz, 2H), 1.54 (s, 3H).

**^13^C NMR (126 MHz, CDCl3)** δ 196.6, 192.6, 172.6, 171.4, 161.5, 144.0, 135.4, 133.0, 128.7, 127.9, 126.6, 120.0, 118.4, 109.6, 104.5, 60.6, 53.1, 52.0, 45.7, 21.2, 14.3.

**HRMS (ESI) m/z**: [M+H]^+^ Calcd for C_23_H_23_NO_5_H^+^394.1649; Found 394.1643.

**Methyl 4-(6-methyl-1-oxo-2,3,4,9-tetrahydro-1H-carbazol-7-yl)-2-oxo-4-phenylbutanoate (3ai)**

From methyl (E)-2-oxo-4-phenylbut-3-enoate (38 mg, 0.2 mmol) and methyl 6-methyl-2,3,4,9-tetrahydro-1H-carbazol-1-one (60 mg, 0.3 mmol), following the general procedure, the title compound (39.6mg, 51%) was obtained as a yellow oil. **R*_f_*** = 0.3 (petroleum ether / ethyl acetate = 5:1).

**^1^H NMR (500 MHz, CDCl3)** δ 8.73 (s, 1H), 7.45 – 7.42 (m, 3H), 7.36 – 7.31 (m, 3H), 7.28 (d, *J* = 7.3 Hz, 1H), 7.21 (d, *J* = 8.5 Hz, 1H), 6.97 (d, *J* = 15.8 Hz, 1H), 6.24 (m, 1H), 3.88 (s, 3H), 3.82 (m, 1H), 3.44 (dd, *J* = 13.1, 4.2 Hz, 1H), 3.16 – 3.11 (m, 1H), 2.94 – 2.87 (m, 1H), 2.44 (s, 3H), 2.39 – 2.35 (m, 1H), 2.31 – 2.25 (m, 1H).

**^13^C NMR (126 MHz, CDCl3)** δ 189.8 175.6, 136.9, 136.3, 131.8, 131.5, 130.1, 129.7, 129.5, 128.8, 128.6, 128.2, 126.9, 126.0, 120.8, 112.4, 58.4, 54.1, 53.5, 25.6, 21.6, 21.1.

**HRMS (ESI) m/z**: [M+H]^+^ Calcd for C_24_H_22_NO_4_H^+^388.1554; Found 388.1553.

**1a** (4.5 mmol, 1.5 equiv), **2a** (3 mmol, 1 equiv), PTS (20 mol%) were dissolved in MeCN (25 mL) under the protection of N_2_ atmosphere.The reaction mixture was stirred at room temperature for 24 hours. The mixture was concentrated under reduced pressure and the residue was purified by flash chromatography on silica gel eluting with petroleum ether/EtOAc (v/v = 10:1 to 4:1) to afford the products **3aa** (yellow oil: 899 mg, 87%).

# 8 References

S1. Tang, X. Z., Tong, L., Liang, H. J.,Liang, J.,Zou, Y., Zhang, X. J., and Yan, M.(2018). Facile synthesis of substituted diaryl sulfones via a [3 + 3] benzannulation strategy. *Org. Biomol. Chem.,* 16, 3560-3563.

S2. Elena De Vita, Peter Schüler, Scott Lovell, Jasmin Lohbeck, Sven Kullmann, Eitan Rabinovich, Amiram Sananes, Bernd Heßling, Veronique Hamon, Niv Papo, Jochen Hess, Edward W. Tate, Nikolas Gunkel, and Aubry K. Miller. (2018). Depsipeptides Featuring a Neutral P1 Are Potent Inhibitors of Kallikrein-Related Peptidase 6 with On-Target Cellular Activity.*J. Med. Chem.* 61, 19, 8859–8874.

# 9 Copies of NMR Spectra for Compounds


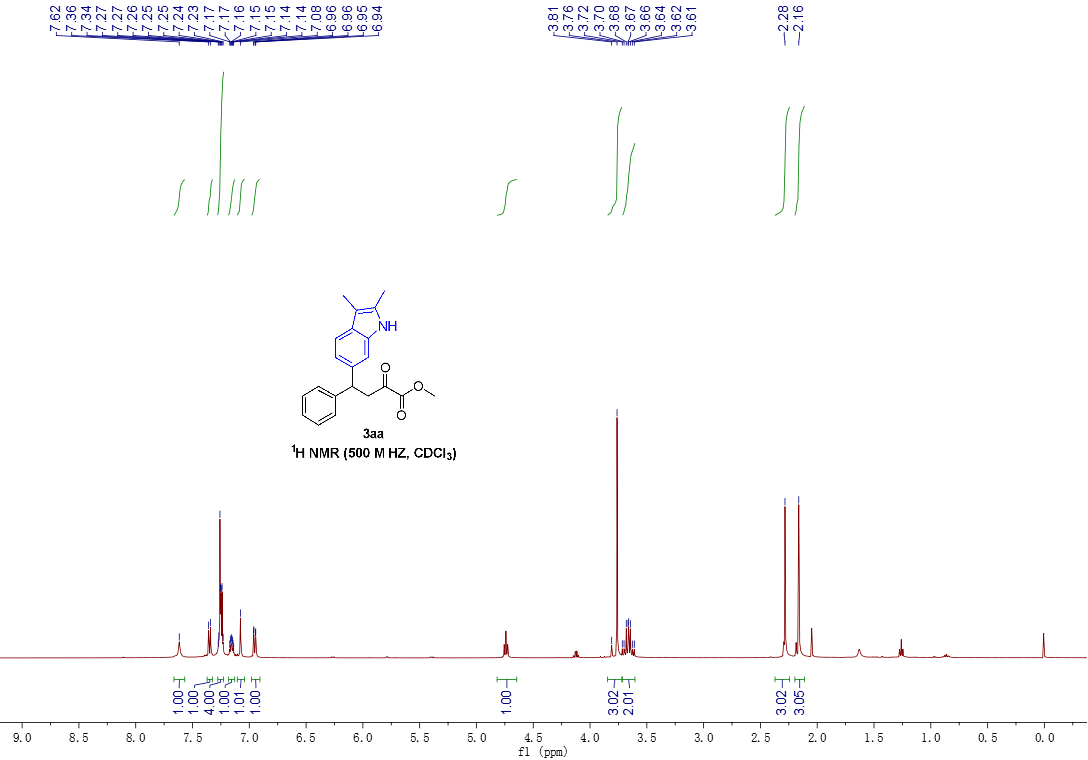
**Figure S3. ^1^H NMR (500 MHz, CDCl_3_) spectrum of 3aa**


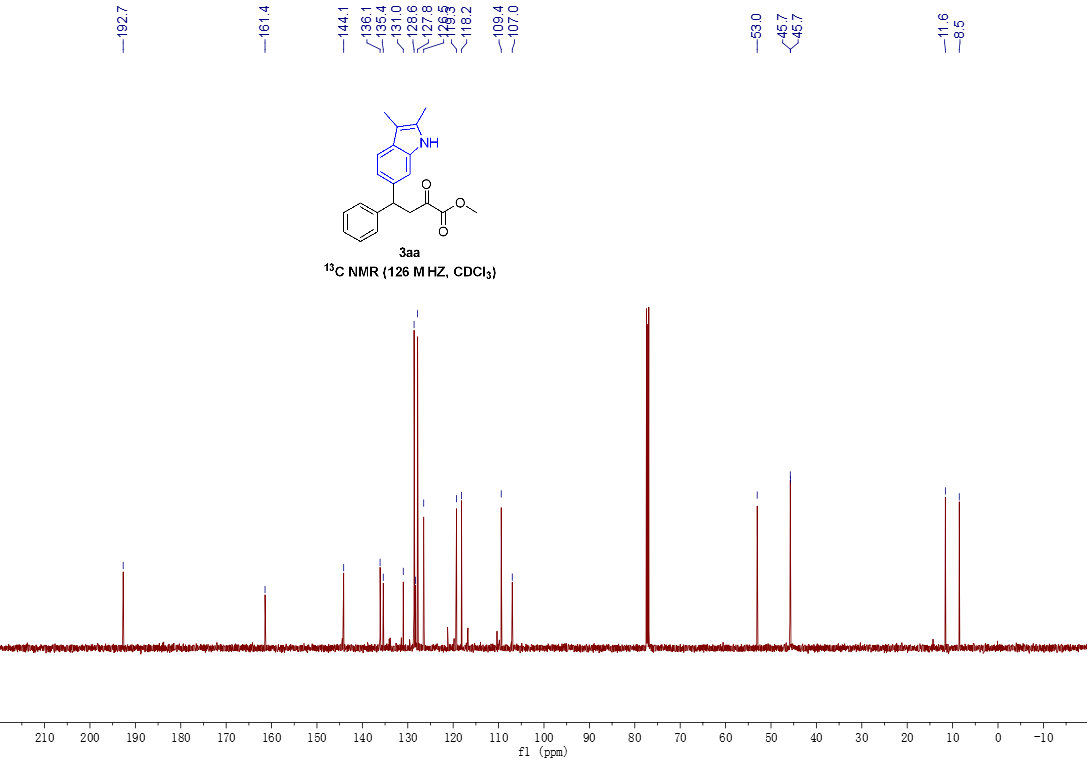


**Figure S4. ^13^C NMR (126 MHz, CDCl_3_) spectrum of 3aa**


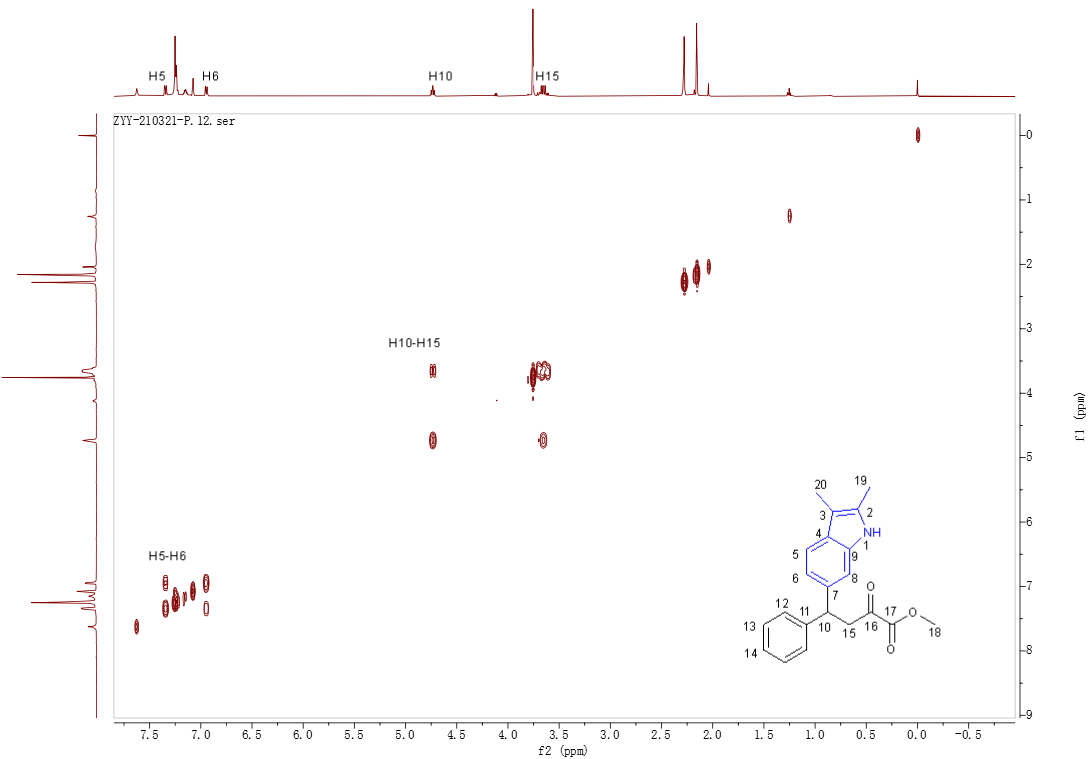


**Figure S5.** **^1^H - ^1^H COSY spectrum of 3aa**


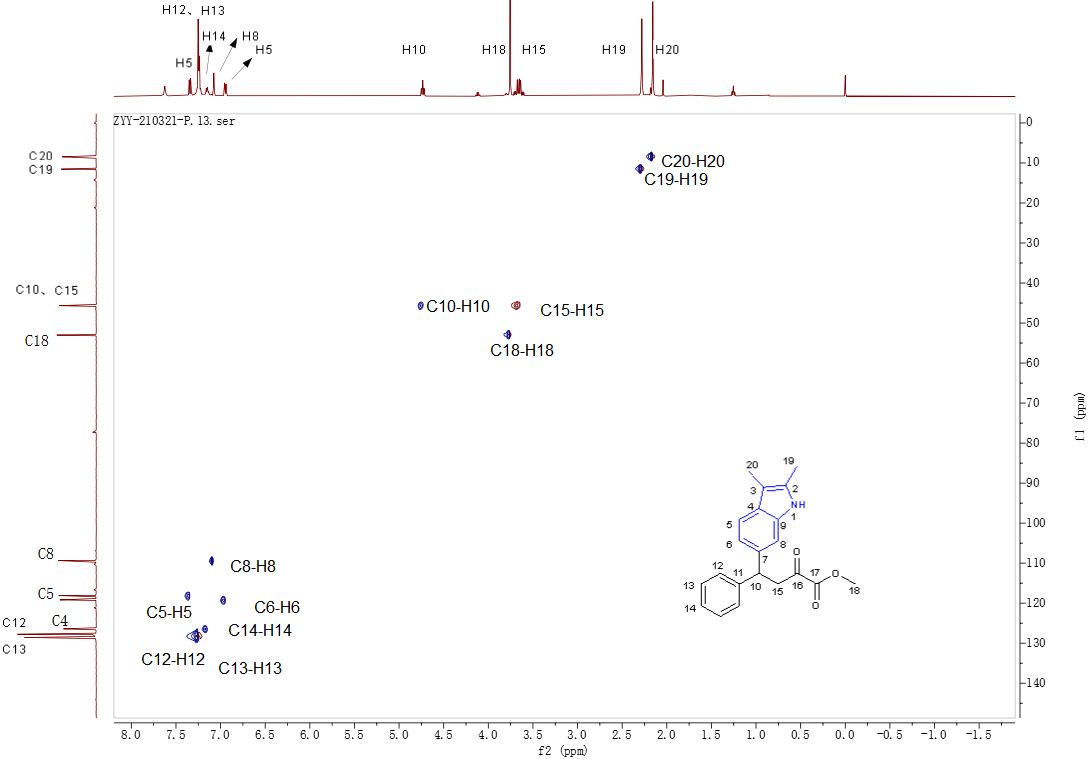


**Figure S6.** **^1^H - ^13^C HSQC spectrum of 3aa**


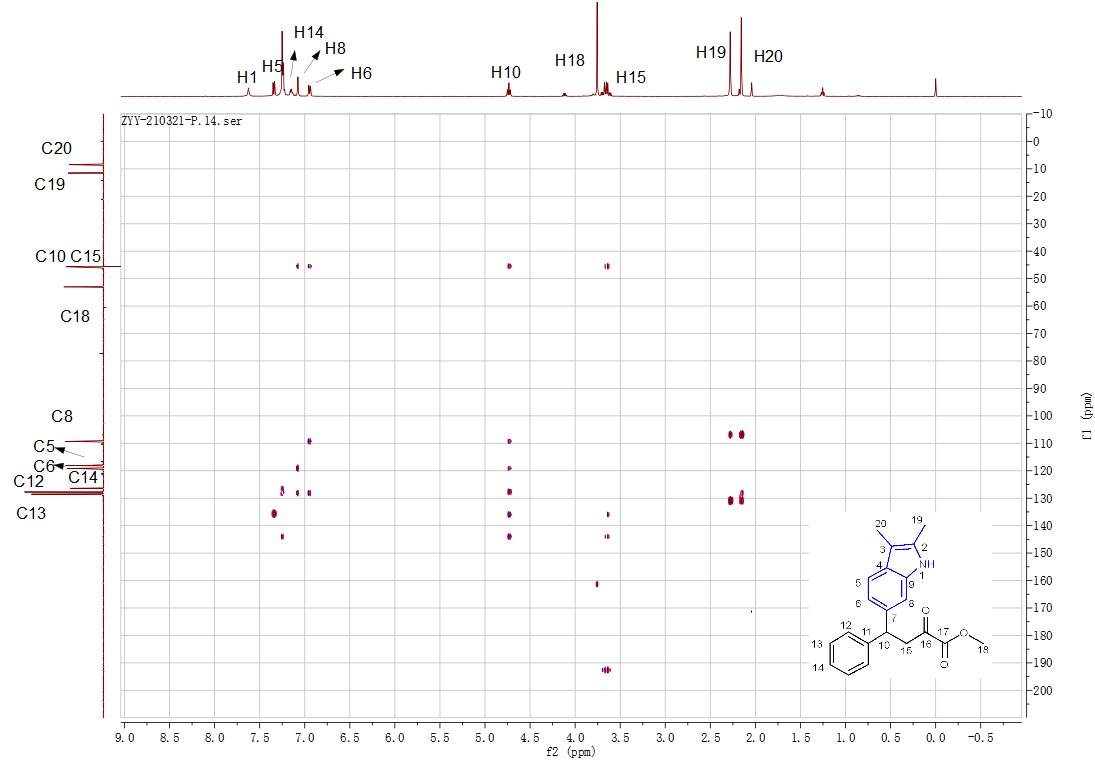


**Figure S7.** **^1^H - ^13^C HMBC spectrum of 3aa**


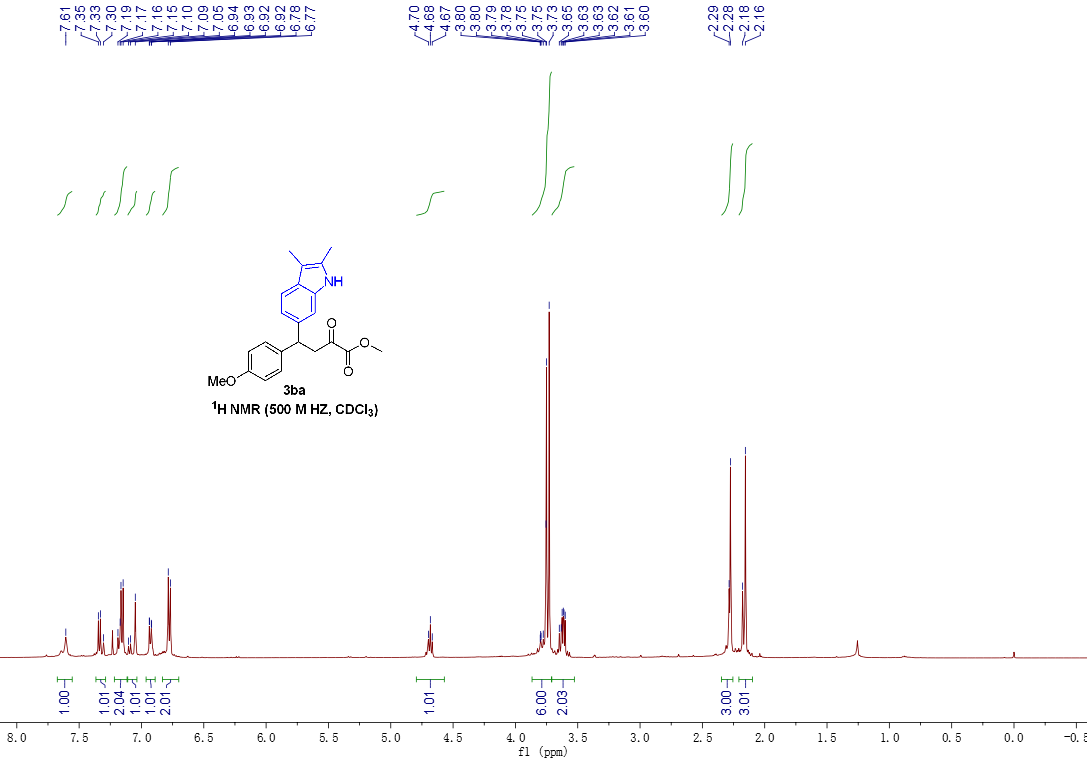


**Figure S8. ^1^H NMR (500 MHz, CDCl_3_) spectrum of 3ba**


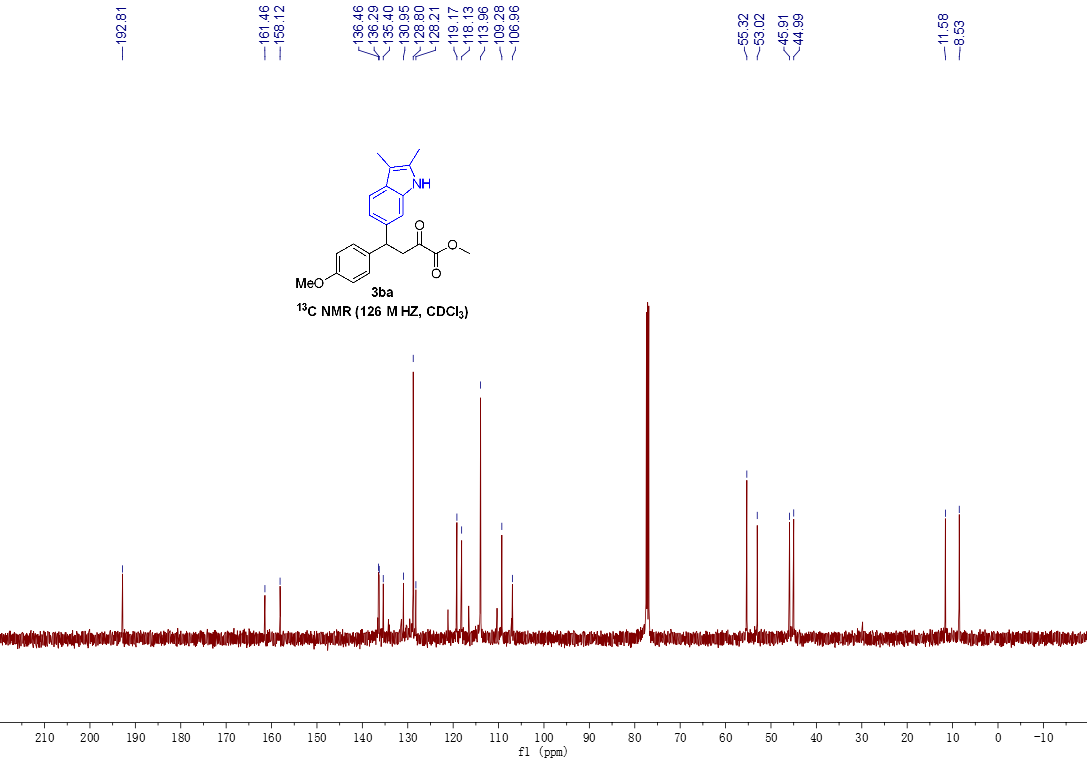


**Figure S9. ^13^C NMR (126 MHz, CDCl_3_) spectrum of 3ba**


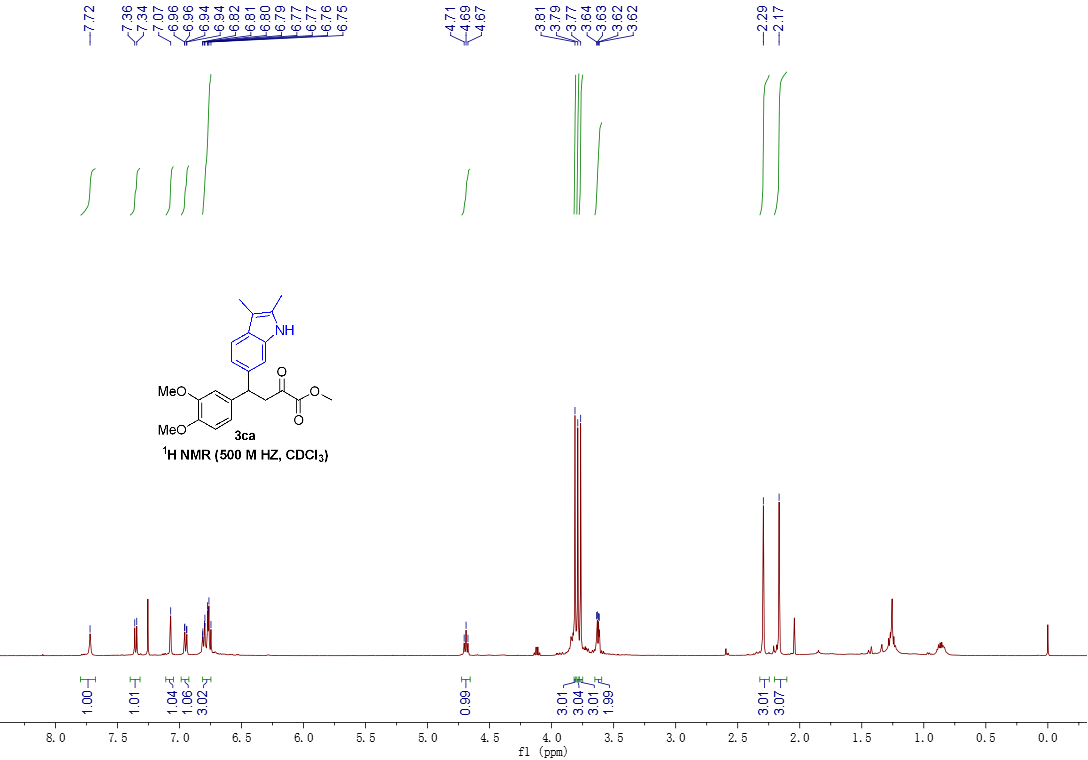
**Figure S10. ^1^H NMR (500 MHz, CDCl_3_) spectrum of 3ca**


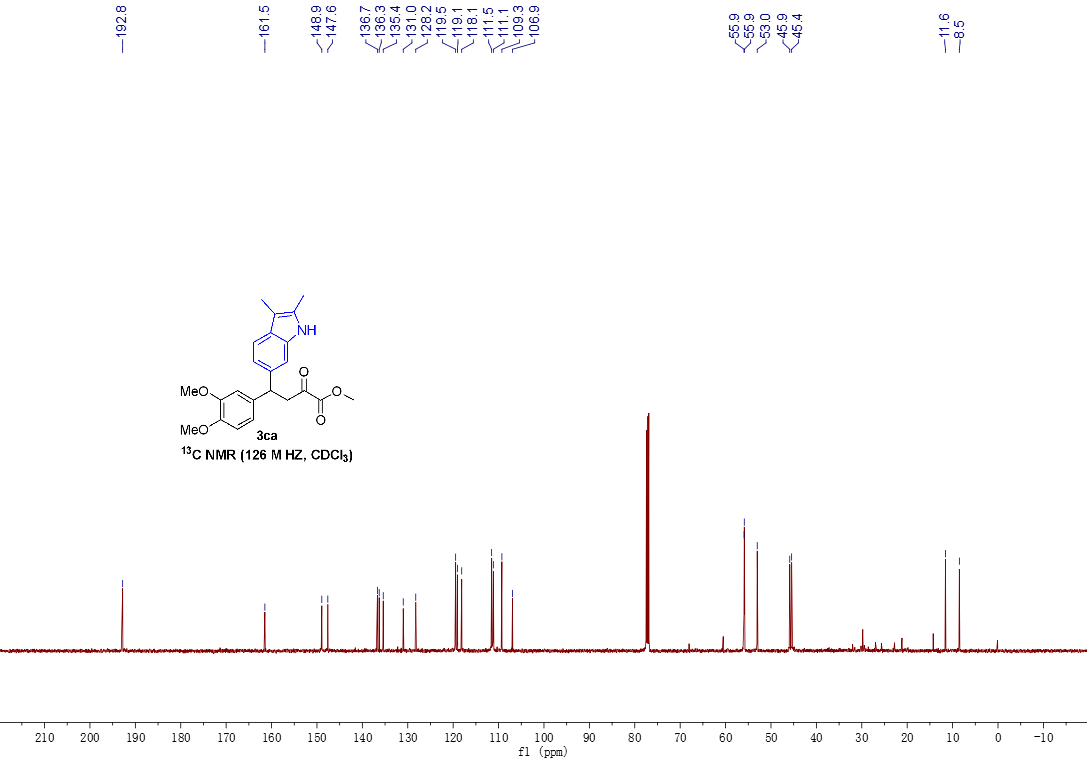
**Figure S11. ^13^C NMR (126 MHz, CDCl_3_) spectrum of 3ca**


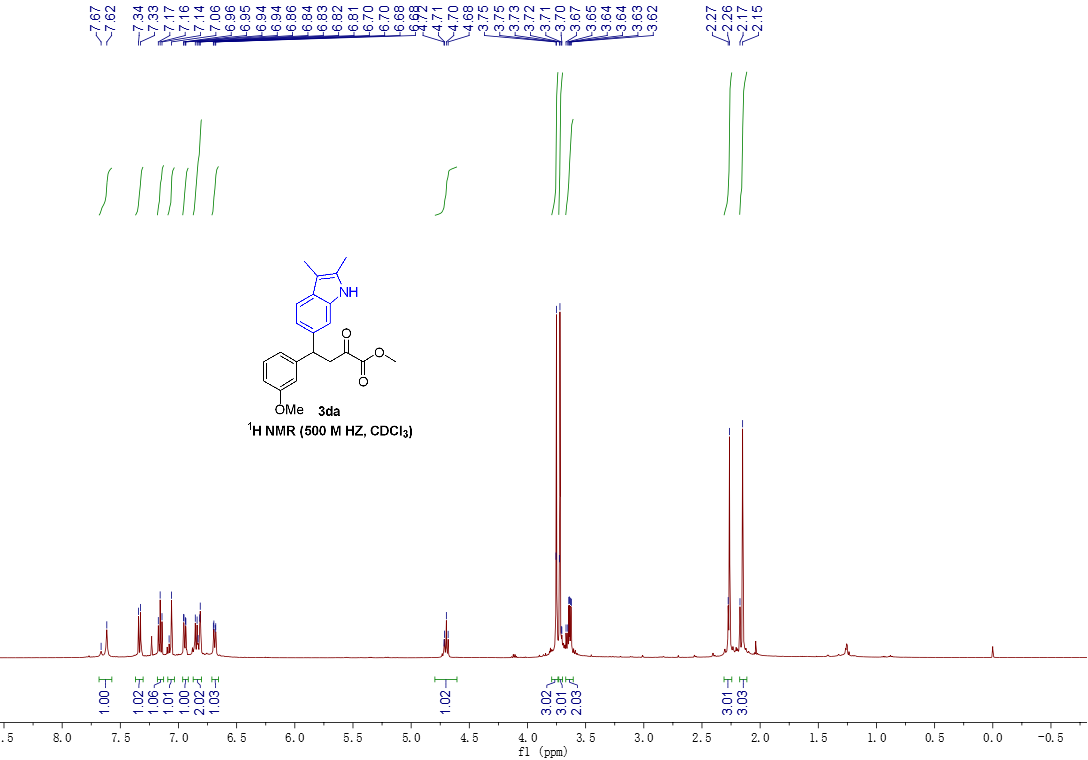
**Figure S12. ^1^H NMR (500 MHz, CDCl_3_) spectrum of 3da**


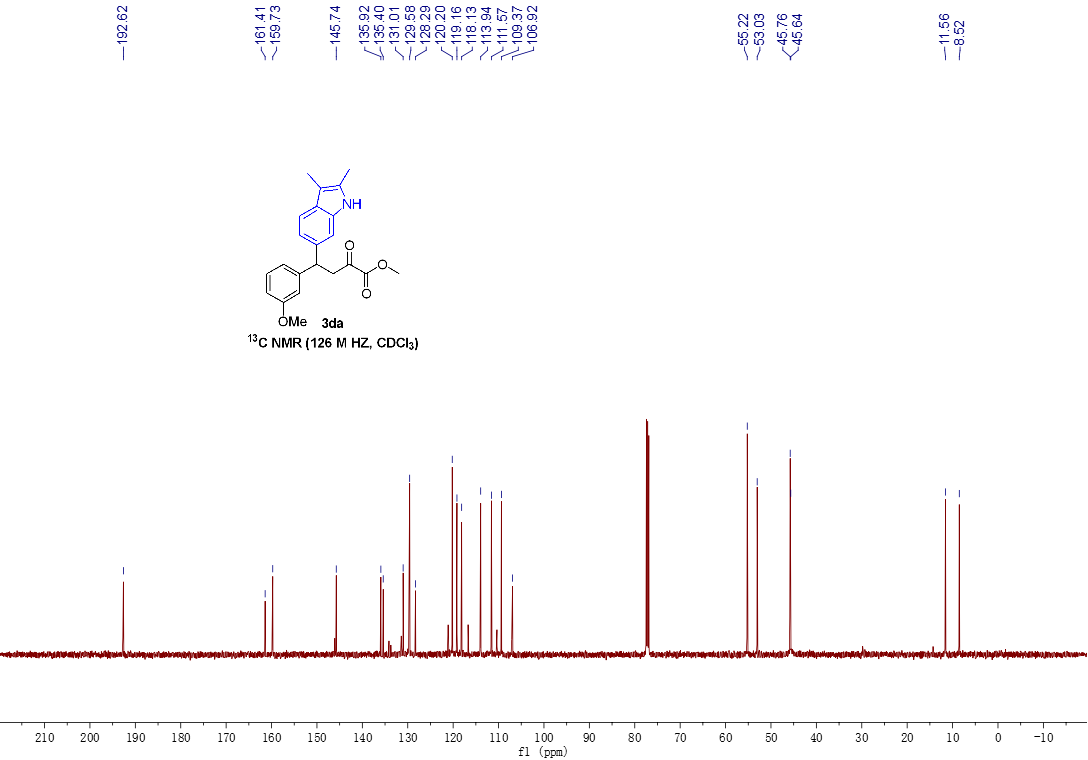
**Figure S13. ^13^C NMR (126 MHz, CDCl_3_) spectrum of 3da**


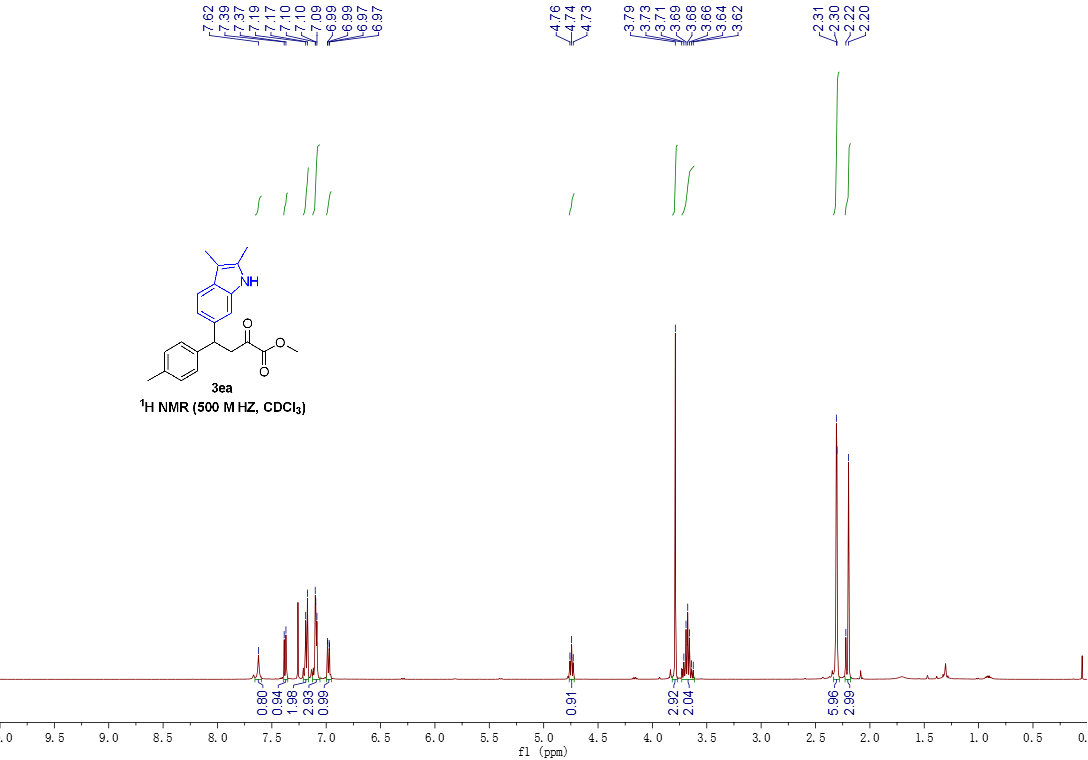
**Figure S14. ^1^H NMR (500 MHz, CDCl_3_) spectrum of 3ea**


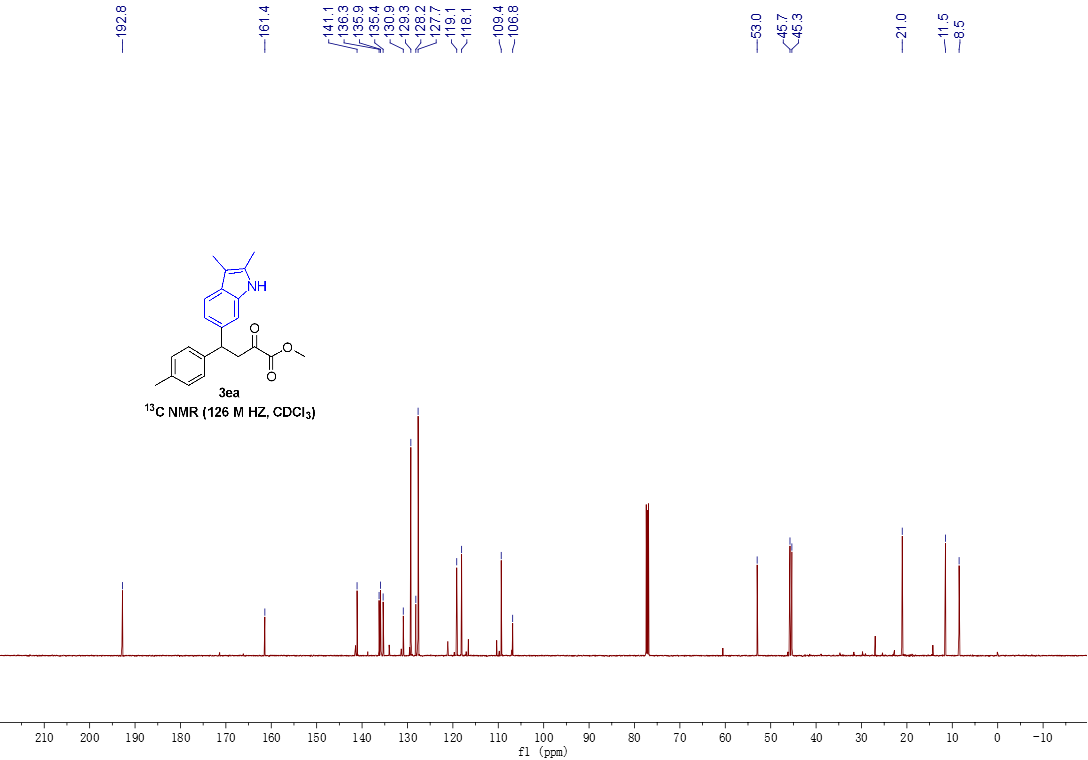
**Figure S15. ^13^C NMR (126 MHz, CDCl_3_) spectrum of 3ea**


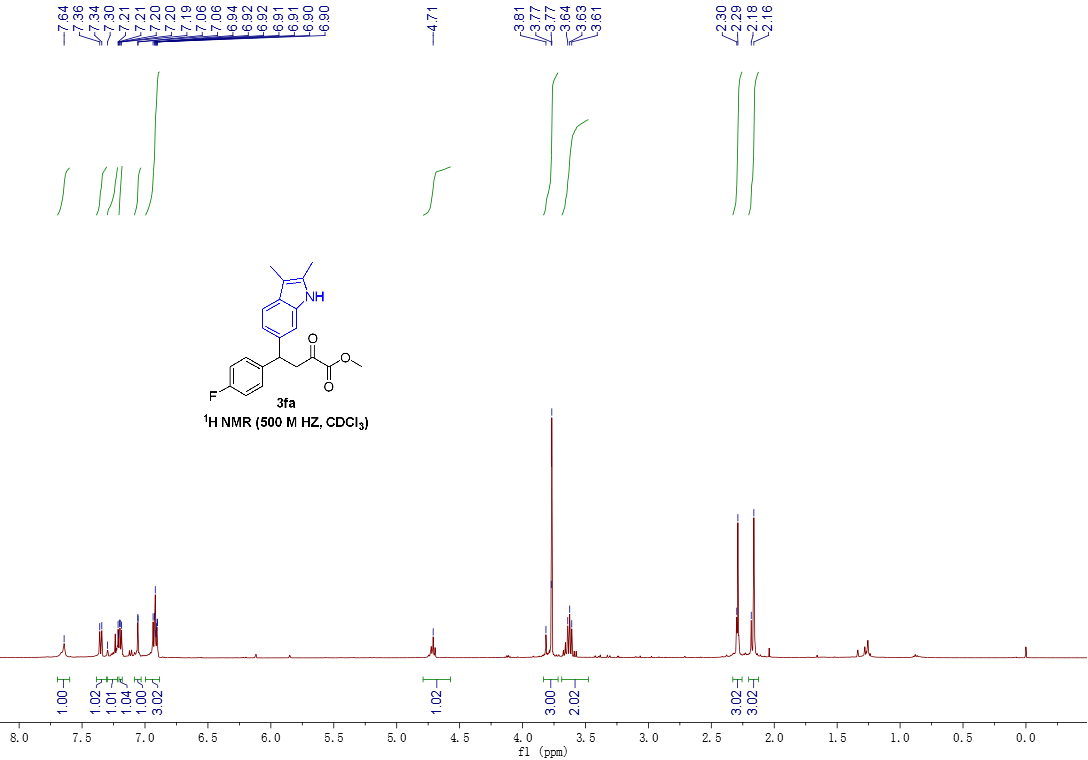
**Figure S16. ^1^H NMR (500 MHz, CDCl_3_) spectrum of 3fa**


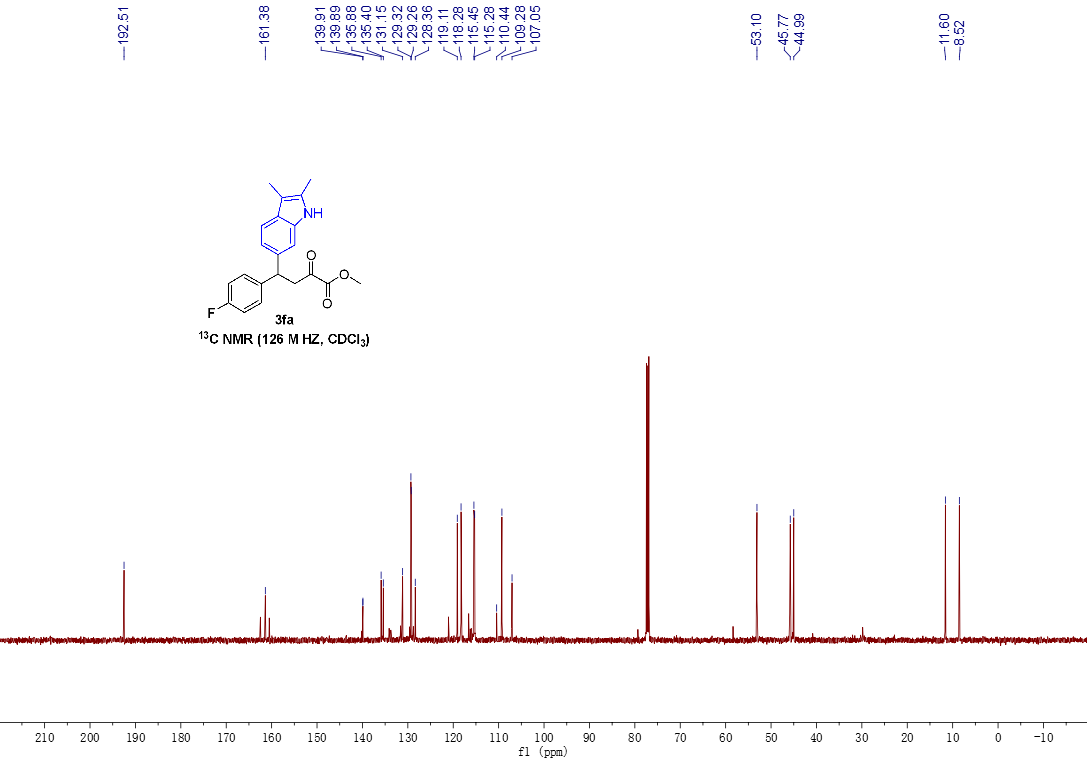


**Figure S17. ^13^C NMR (126 MHz, CDCl_3_) spectrum of 3fa**


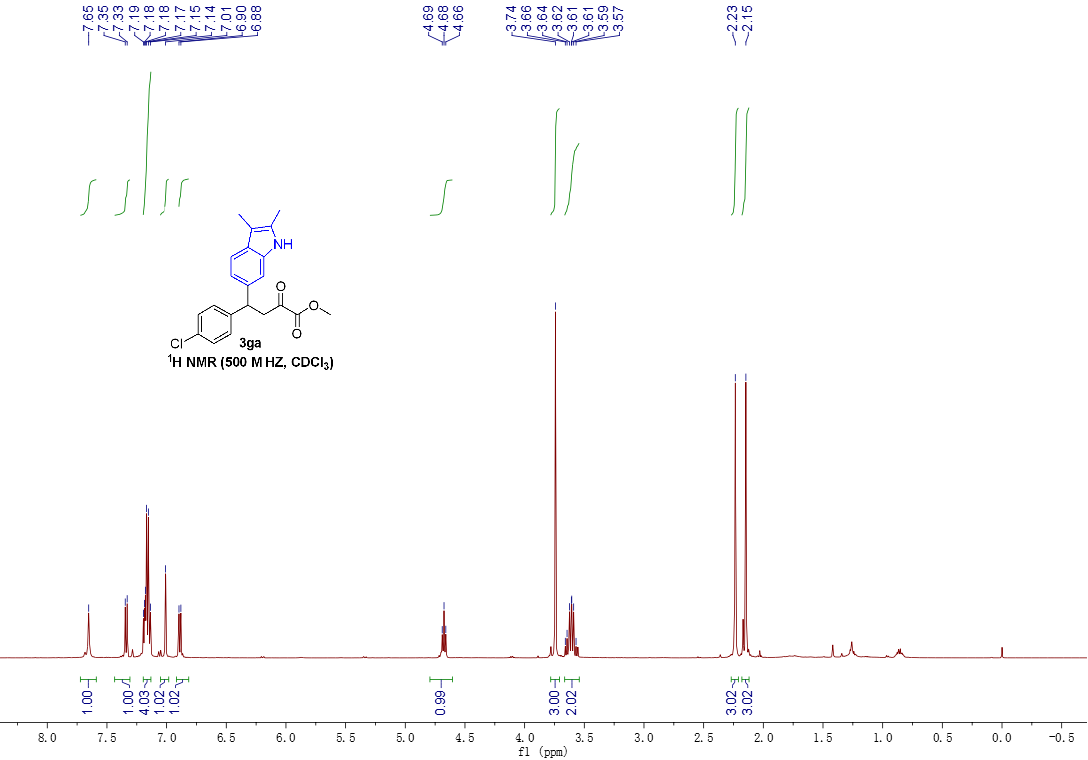
**Figure S18. ^1^H NMR (500 MHz, CDCl_3_) spectrum of 3ga**


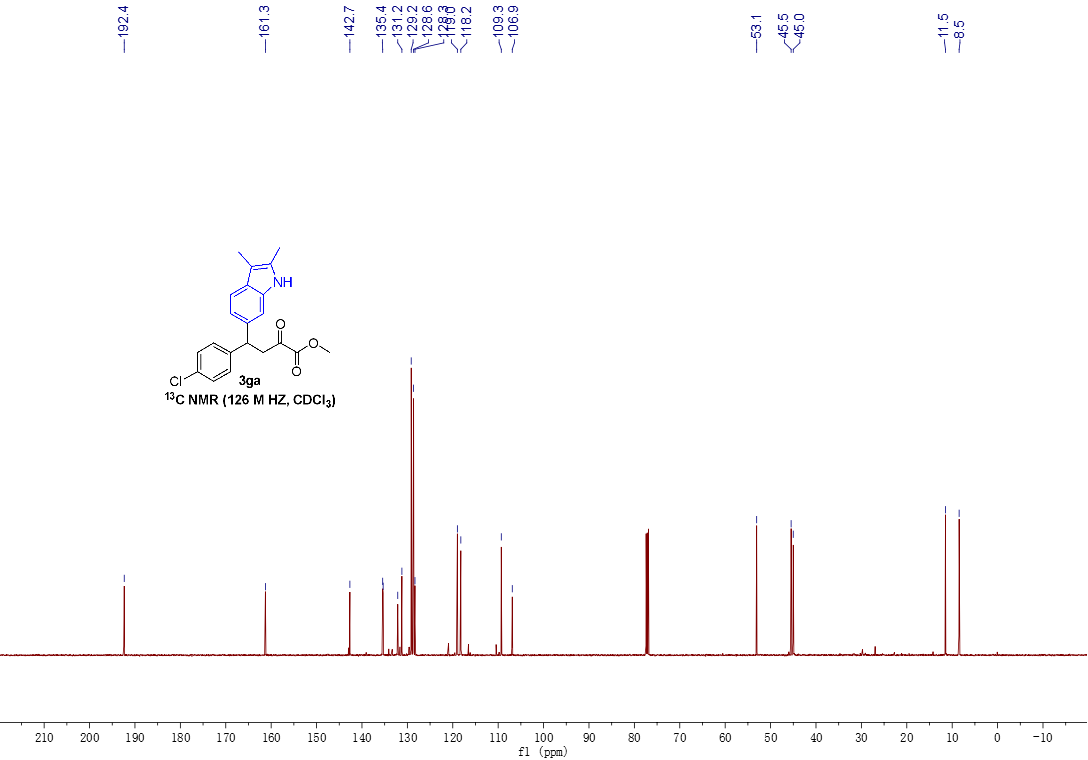


**Figure S19. ^13^C NMR (126 MHz, CDCl_3_) spectrum of 3ga**


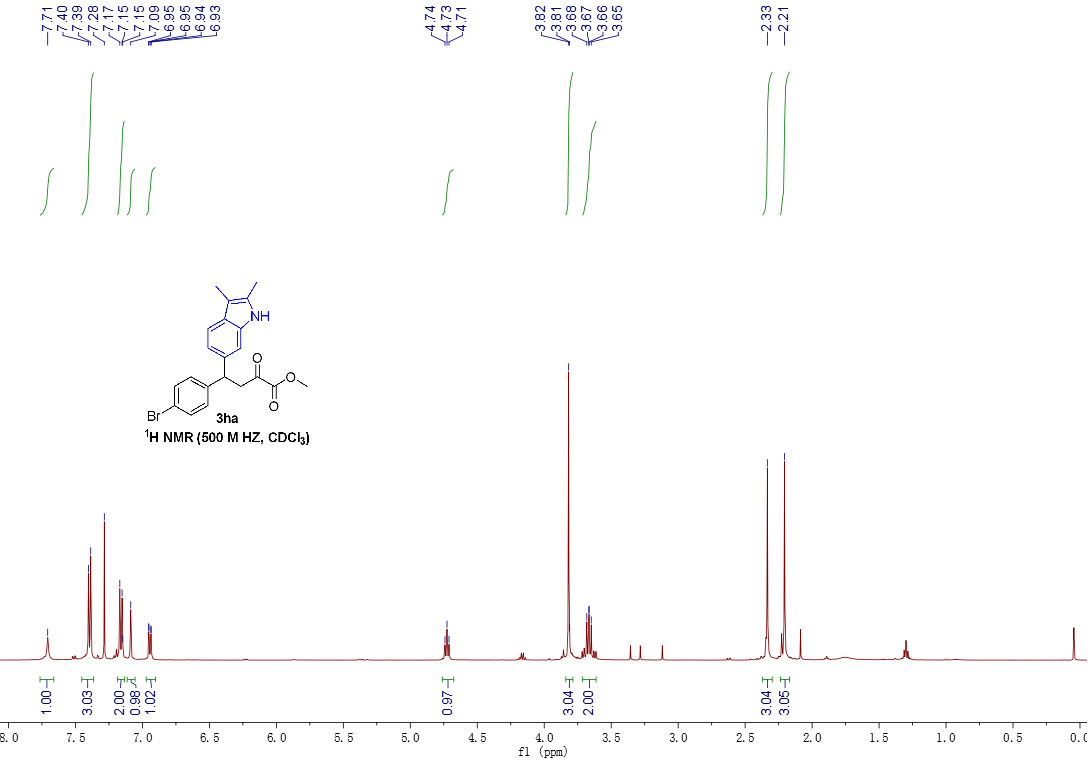


**Figure S20. ^1^H NMR (500 MHz, CDCl_3_) spectrum of 3ha**


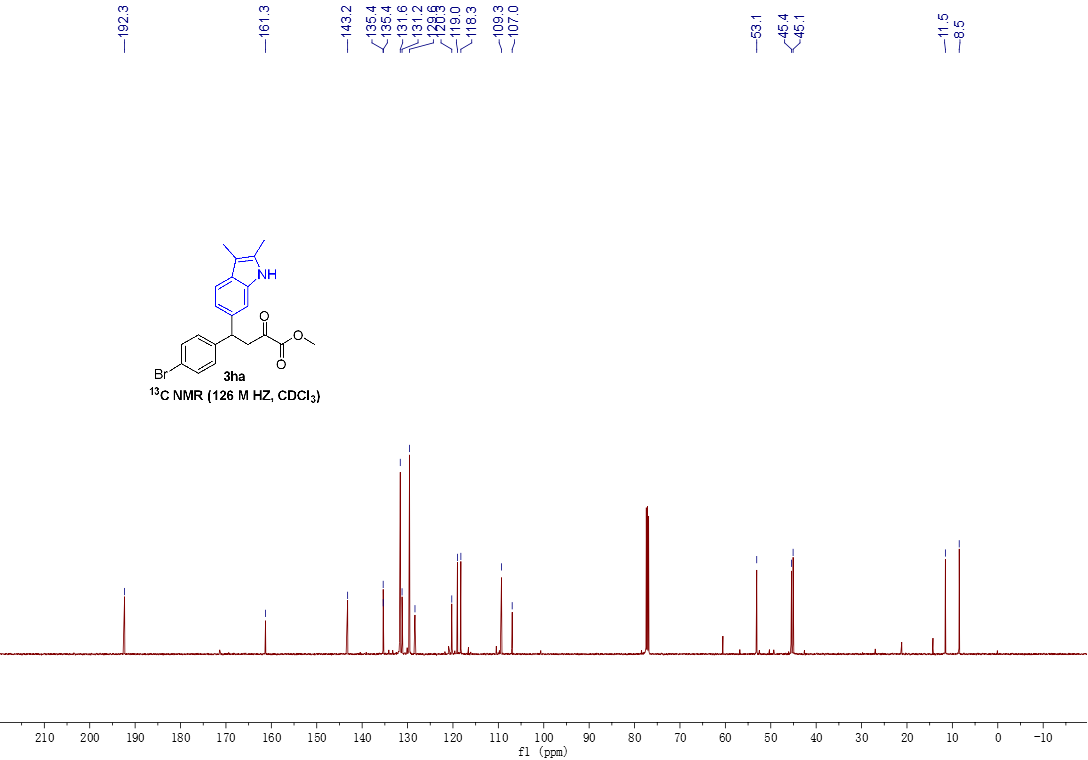


**Figure S21. ^13^C NMR (126 MHz, CDCl_3_) spectrum of 3ha**


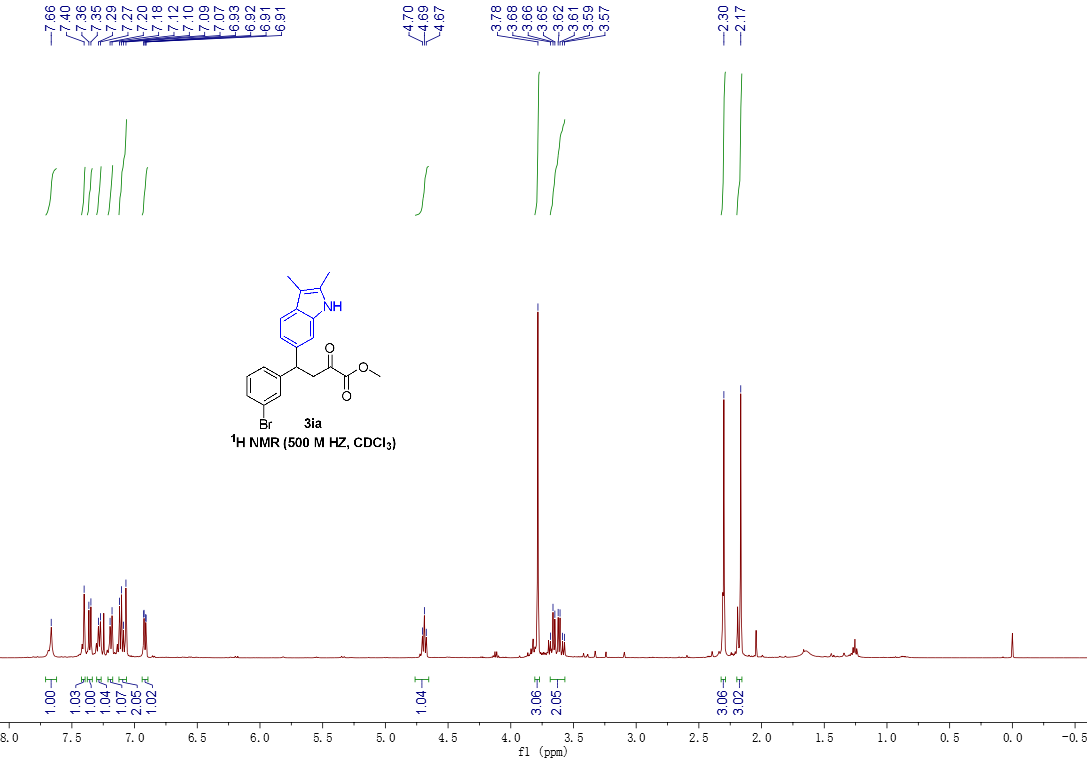
**Figure S22. ^1^H NMR (500 MHz, CDCl_3_) spectrum of 3ia**


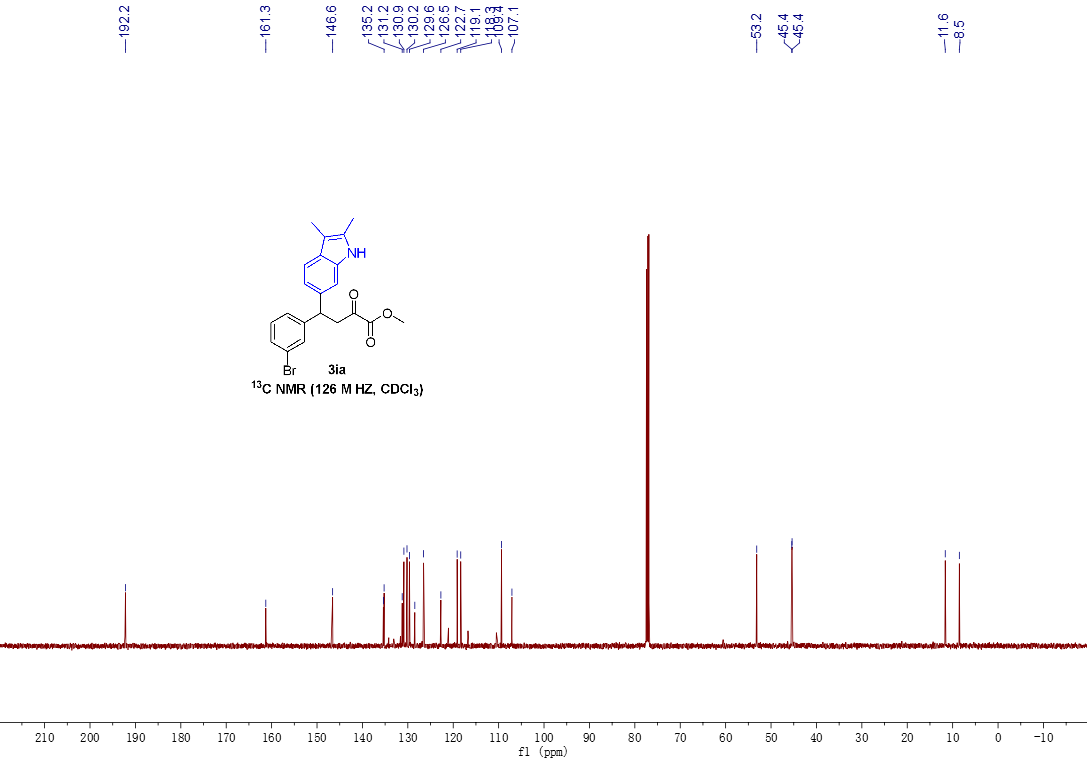
**Figure S23. ^13^C NMR (126 MHz, CDCl_3_) spectrum of 3ia**


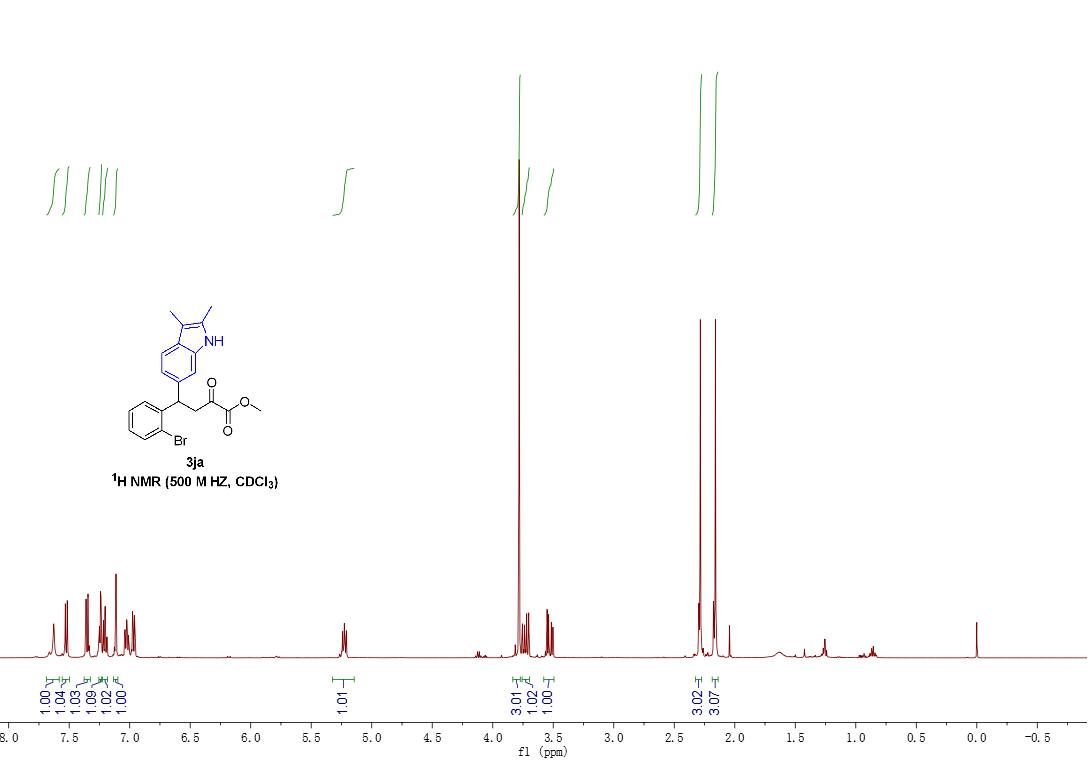


**Figure S24. ^1^H NMR (500 MHz, CDCl_3_) spectrum of 3ja**


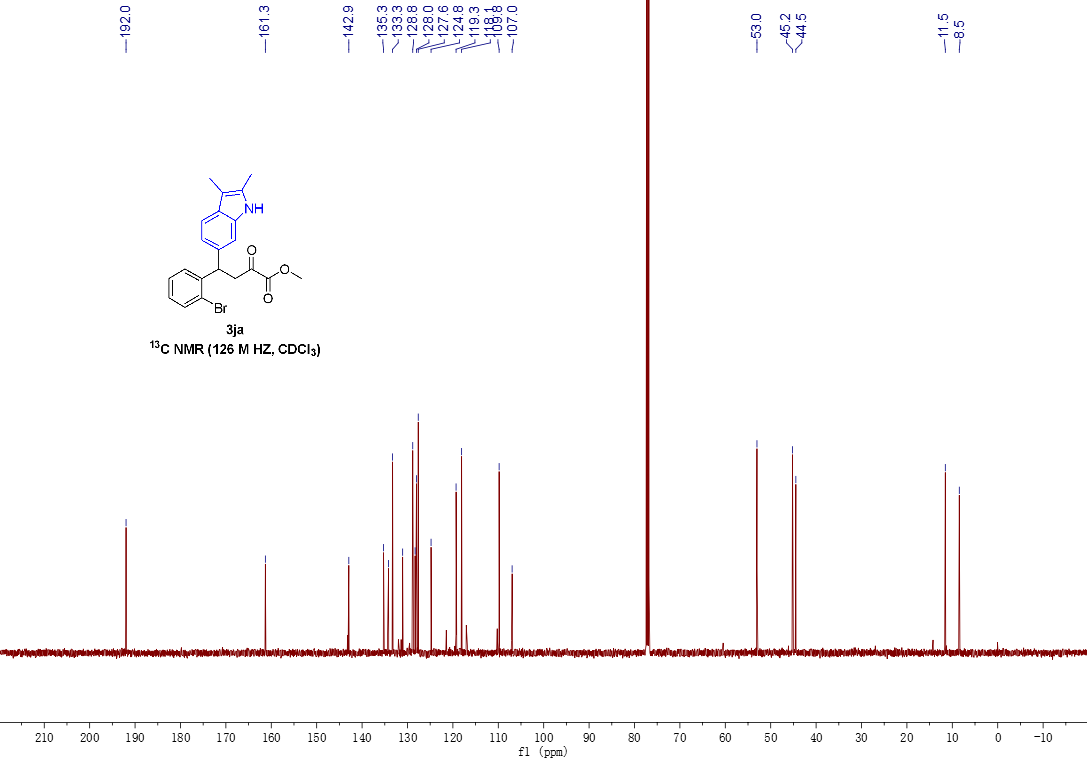


**Figure S25. ^13^C NMR (126 MHz, CDCl_3_) spectrum of 3ja**


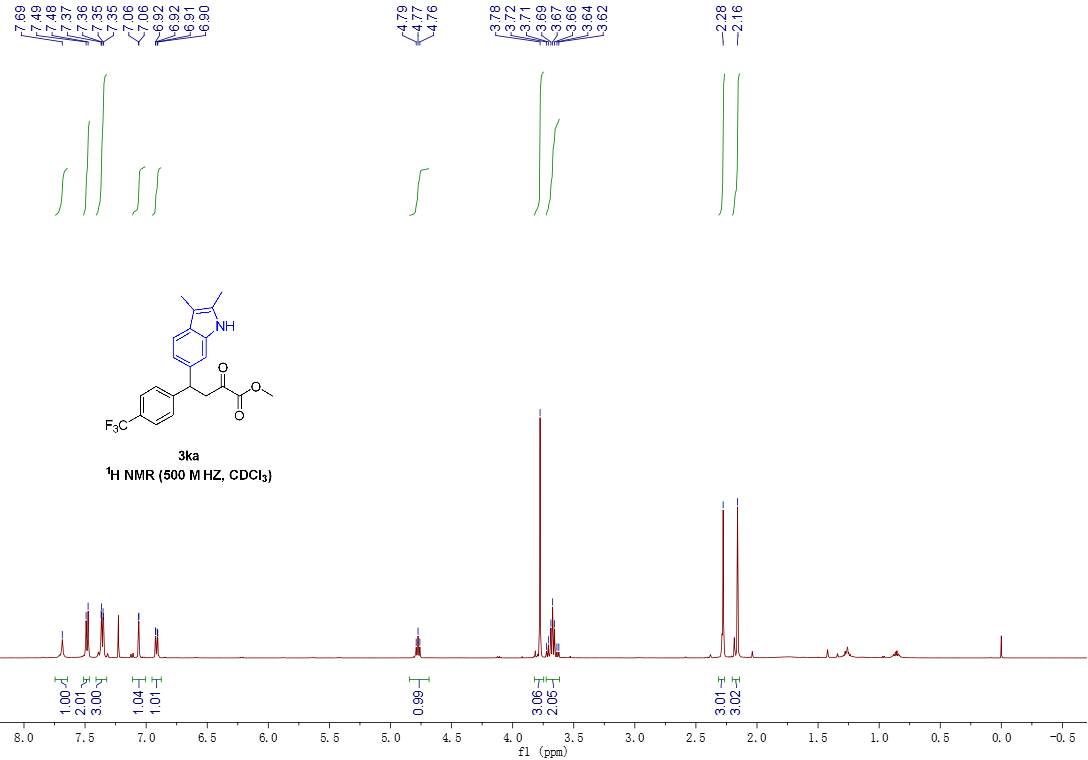


**Figure S26. ^1^H NMR (500 MHz, CDCl_3_) spectrum of 3ka**


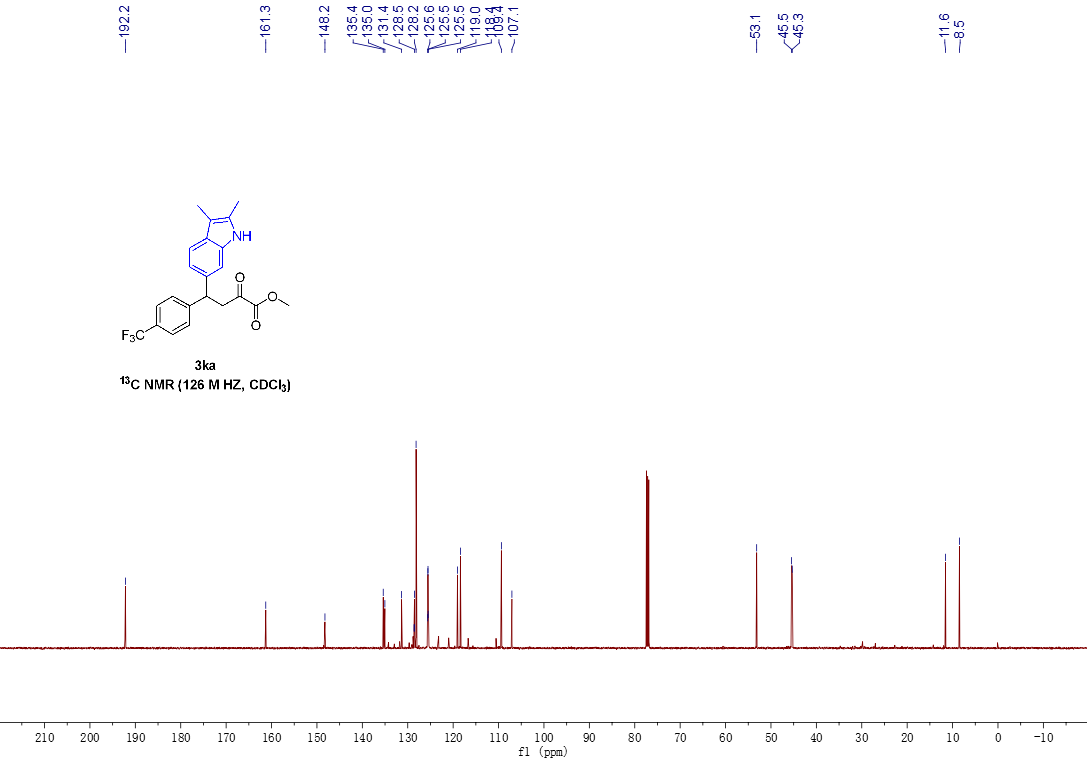


**Figure S27. ^13^C NMR (126 MHz, CDCl_3_) spectrum of 3ka**


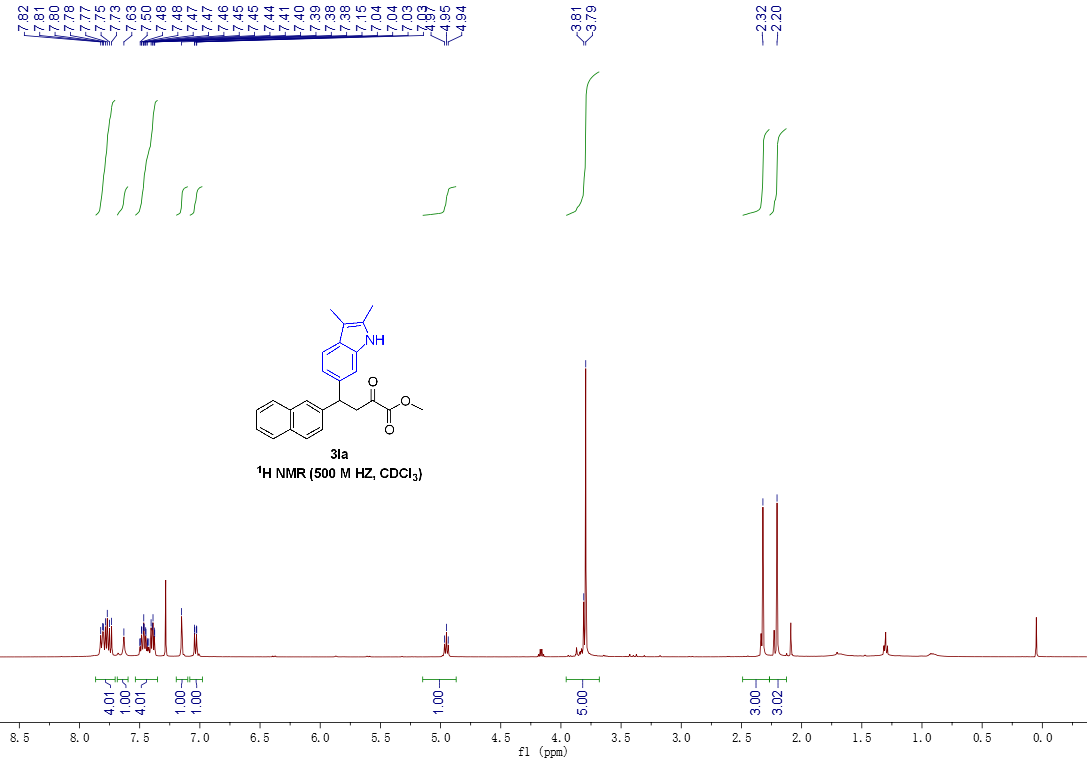
**Figure S28. ^1^H NMR (500 MHz, CDCl_3_) spectrum of 3la**


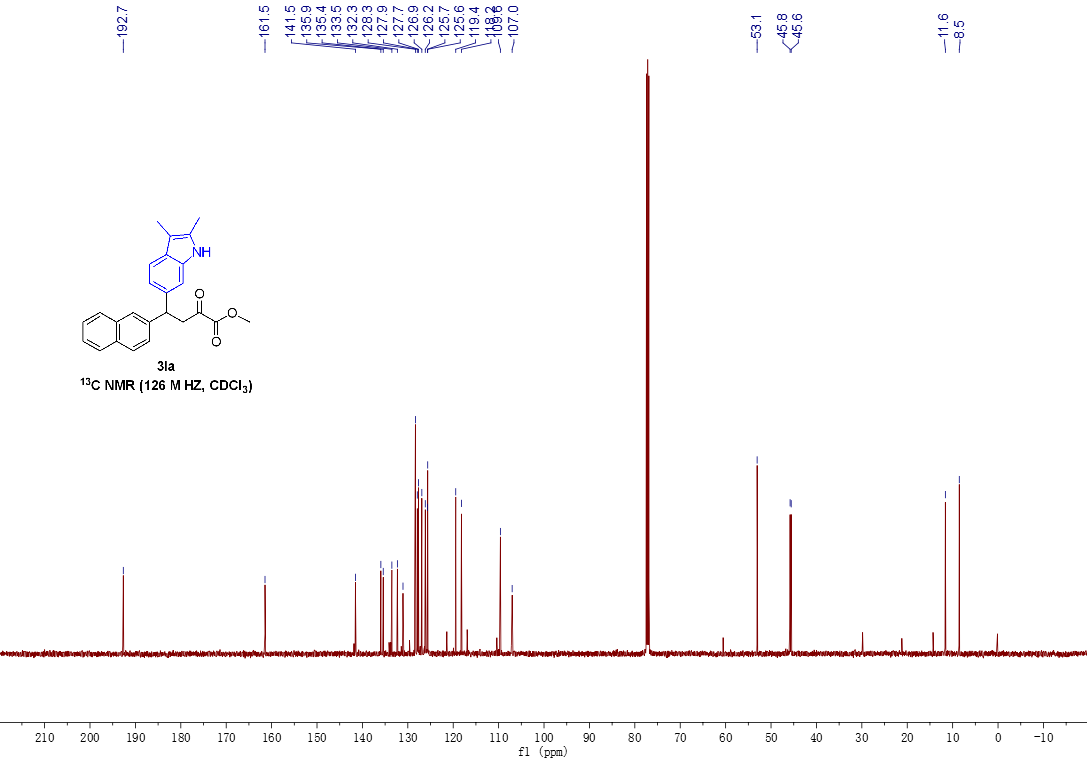
**Figure S29. ^13^C NMR (126 MHz, CDCl_3_) spectrum of 3la**


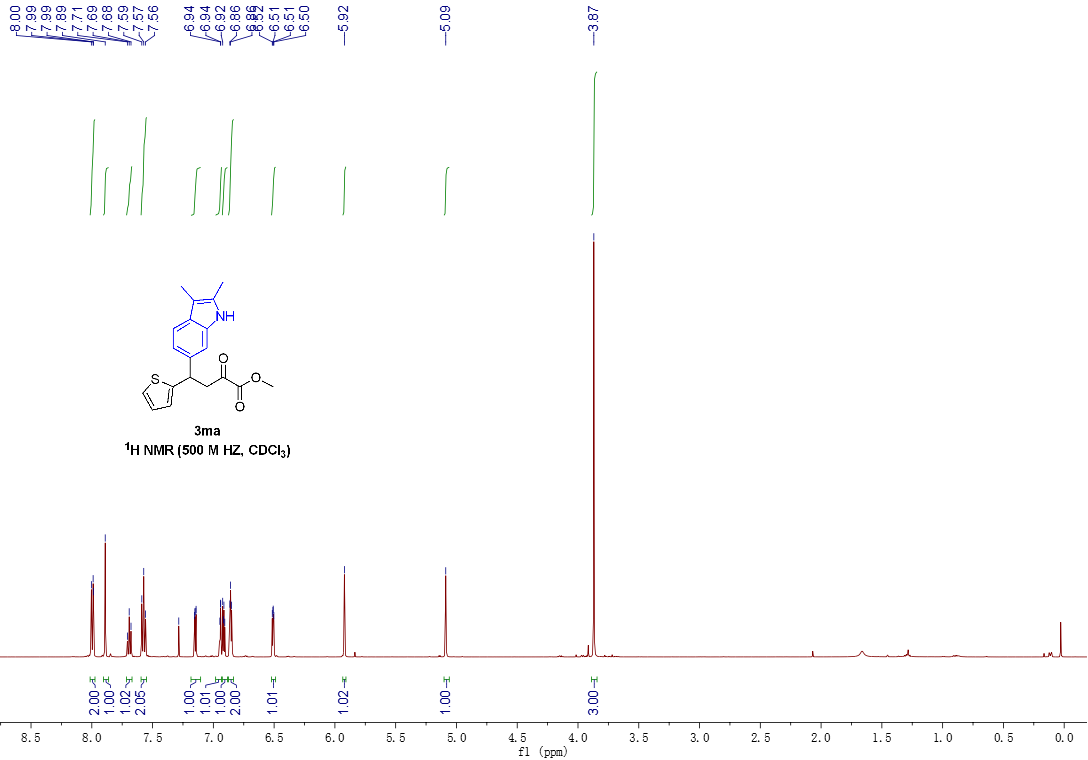
**Figure S30. ^1^H NMR (500 MHz, CDCl_3_) spectrum of 3ma**


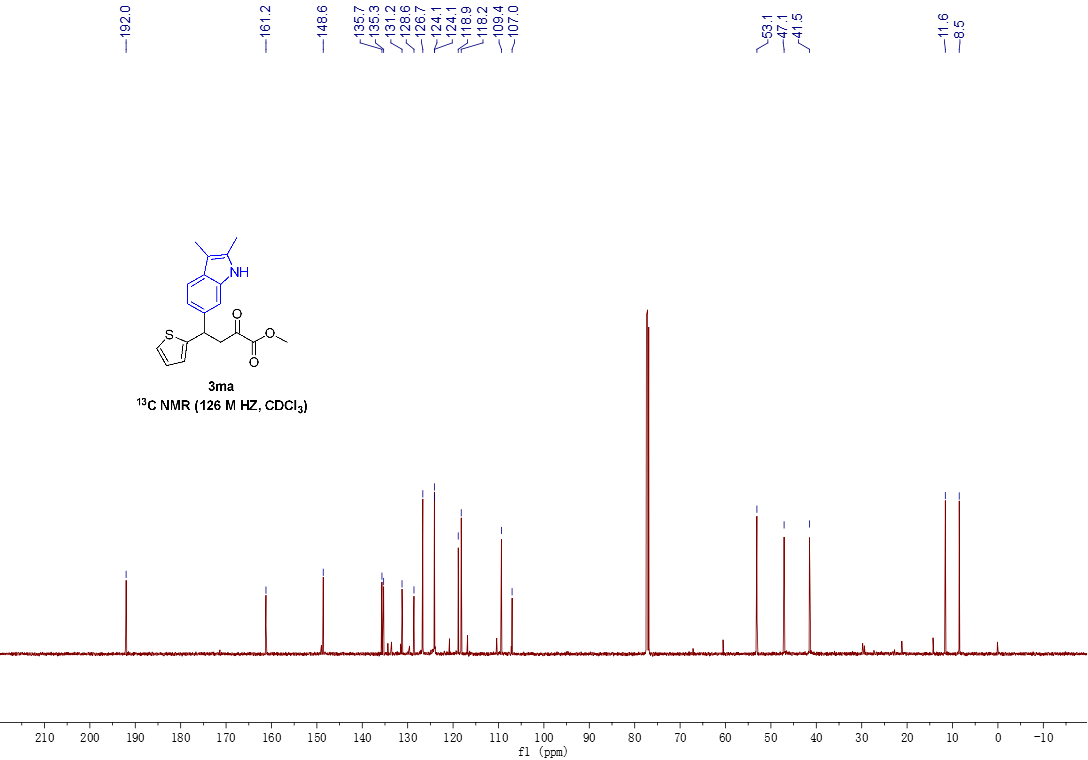


**Figure S31. ^13^C NMR (126 MHz, CDCl_3_) spectrum of 3ma**


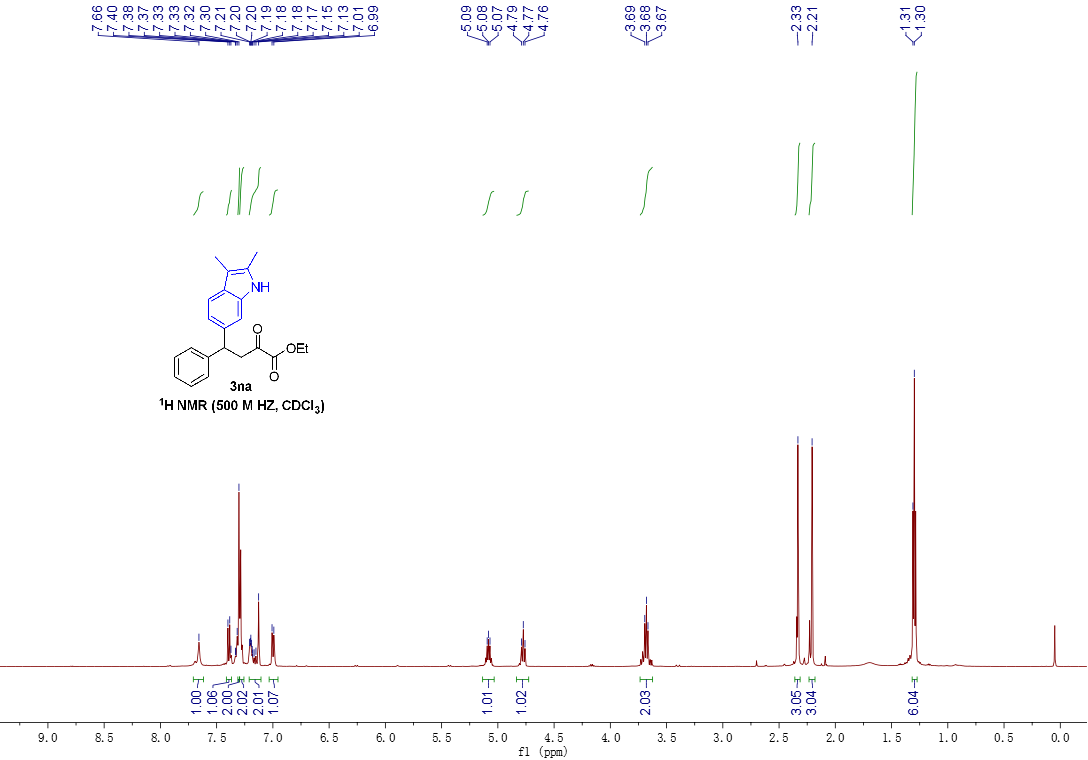
**Figure S32. ^1^H NMR (500 MHz, CDCl_3_) spectrum of 3na**


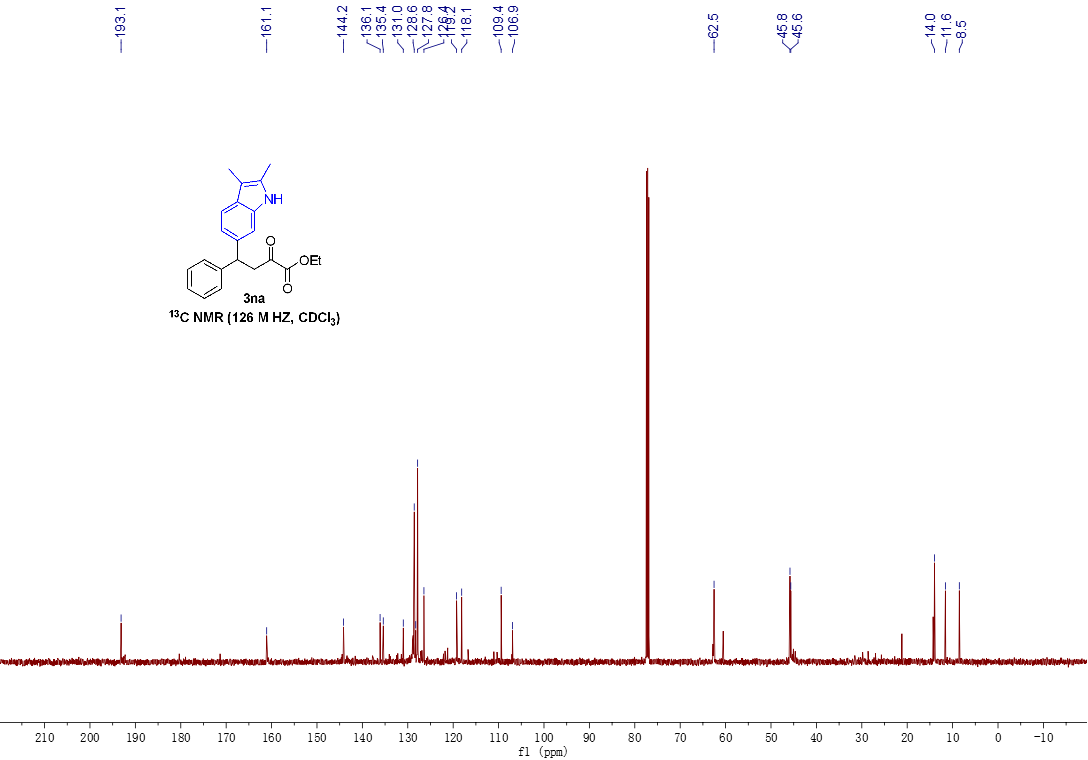


**Figure S33. ^13^C NMR (126 MHz, CDCl_3_) spectrum of 3na**


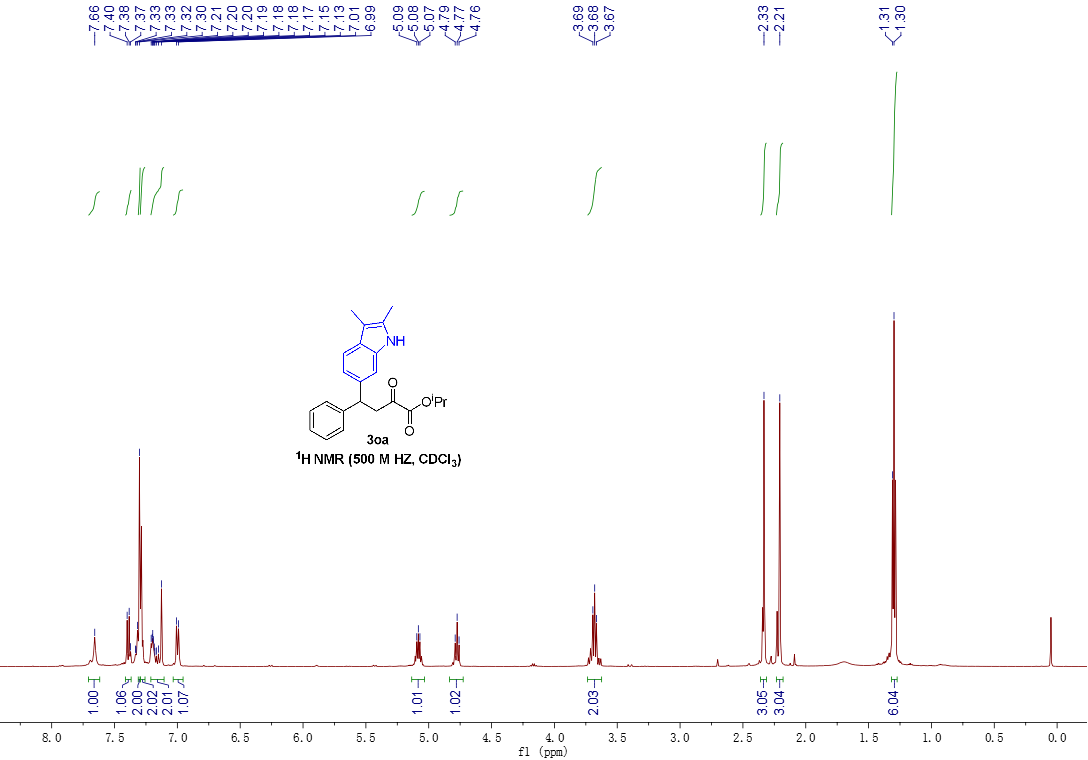


**Figure S34. ^1^H NMR (500 MHz, CDCl_3_) spectrum of 3oa**


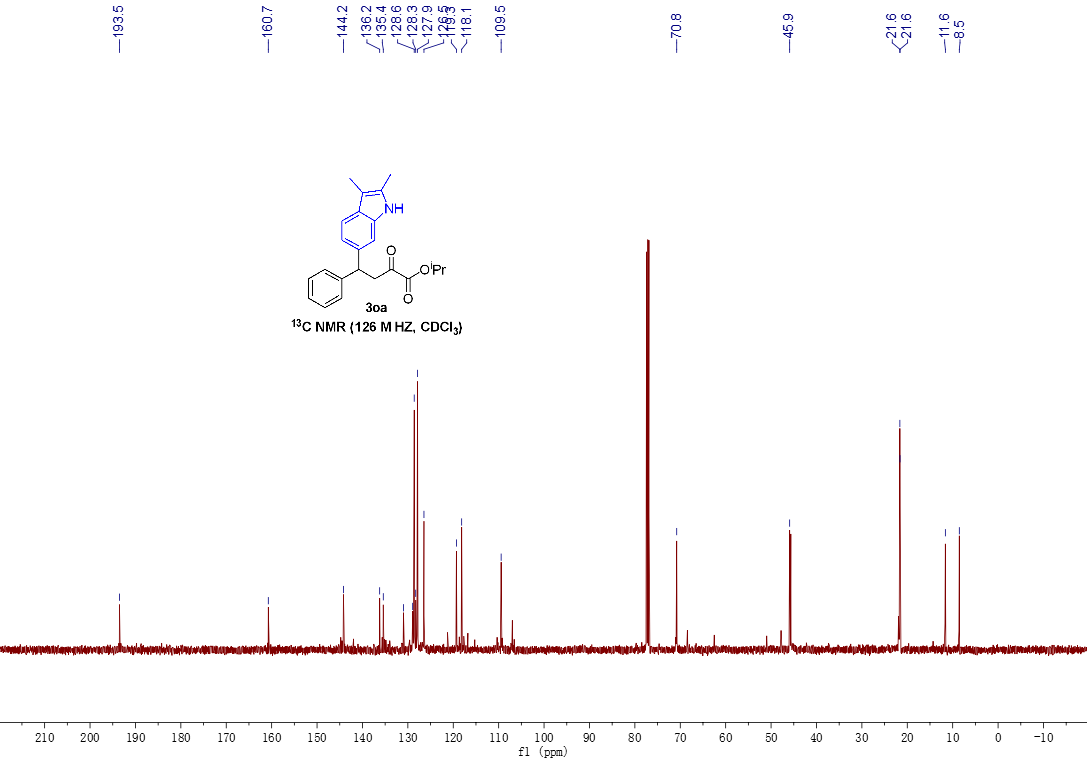


**Figure S35. ^13^C NMR (126 MHz, CDCl_3_) spectrum of 3oa**


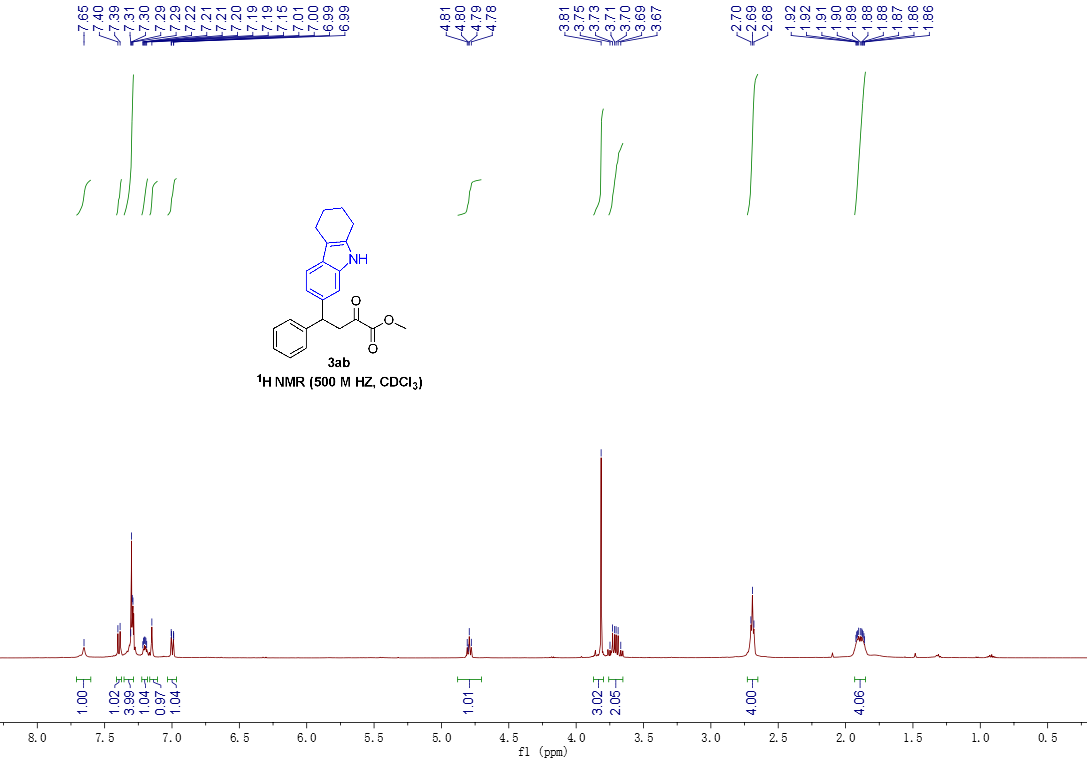


**Figure S36. ^1^H NMR (500 MHz, CDCl_3_) spectrum of 3ab**


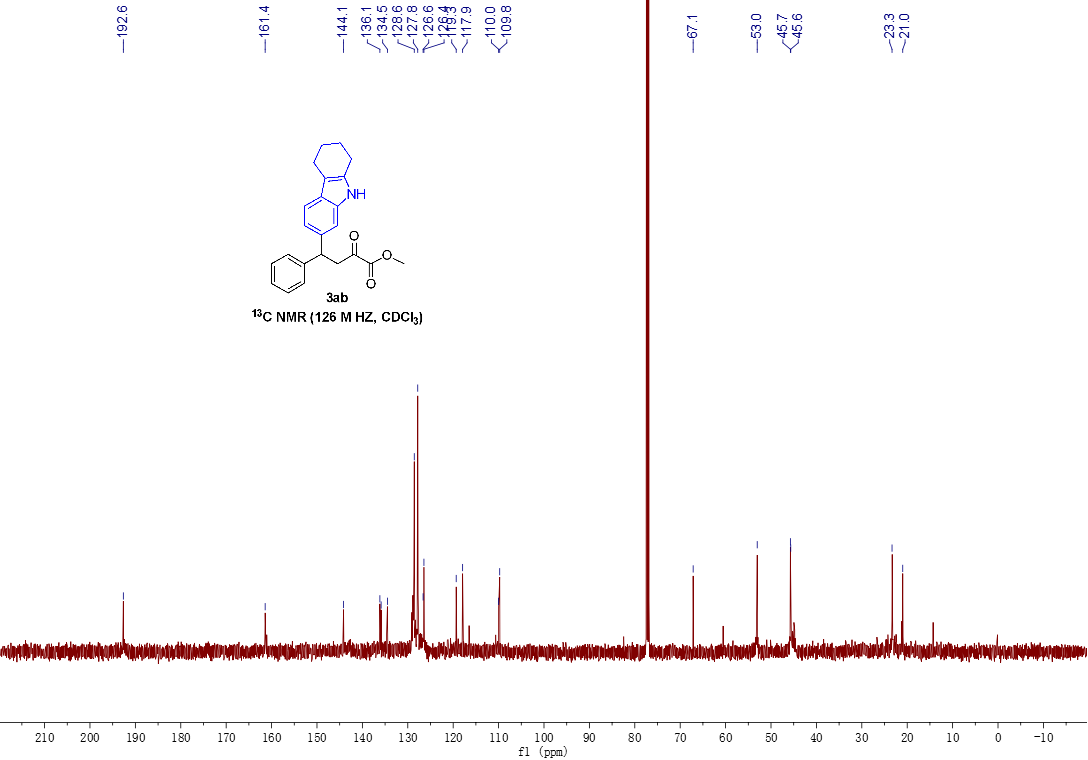
**Figure S37. ^13^C NMR (126 MHz, CDCl_3_) spectrum of 3ab**


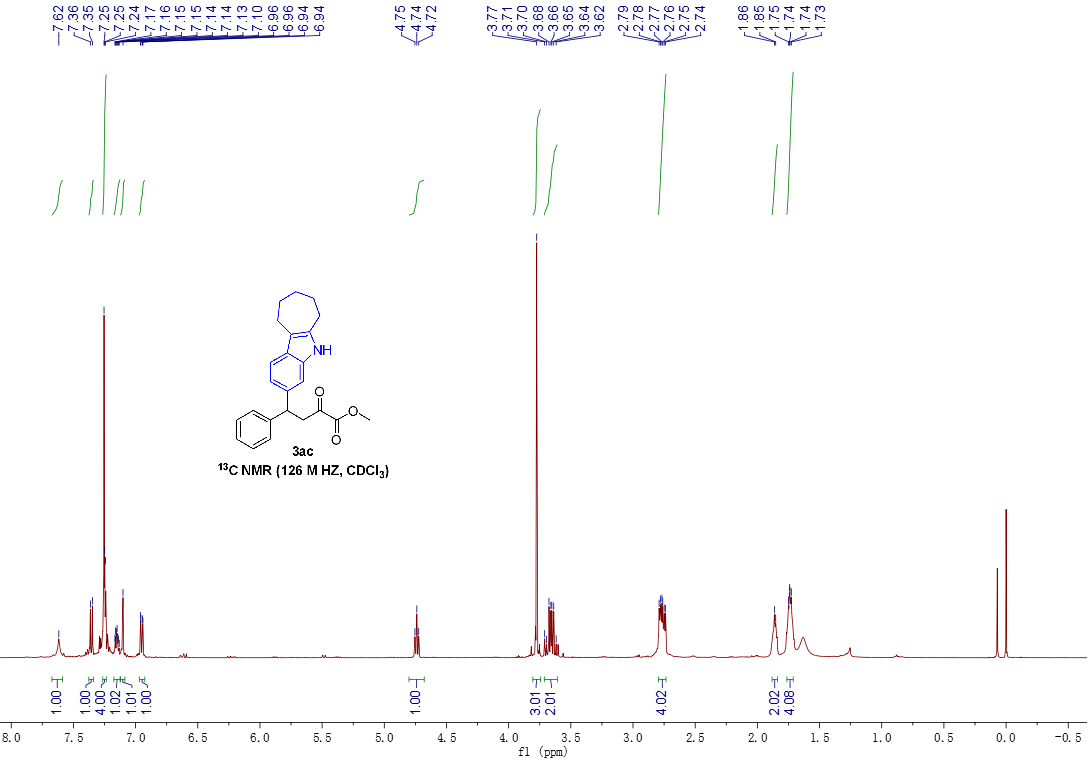


**Figure S38. ^1^H NMR (500 MHz, CDCl_3_) spectrum of 3ac**


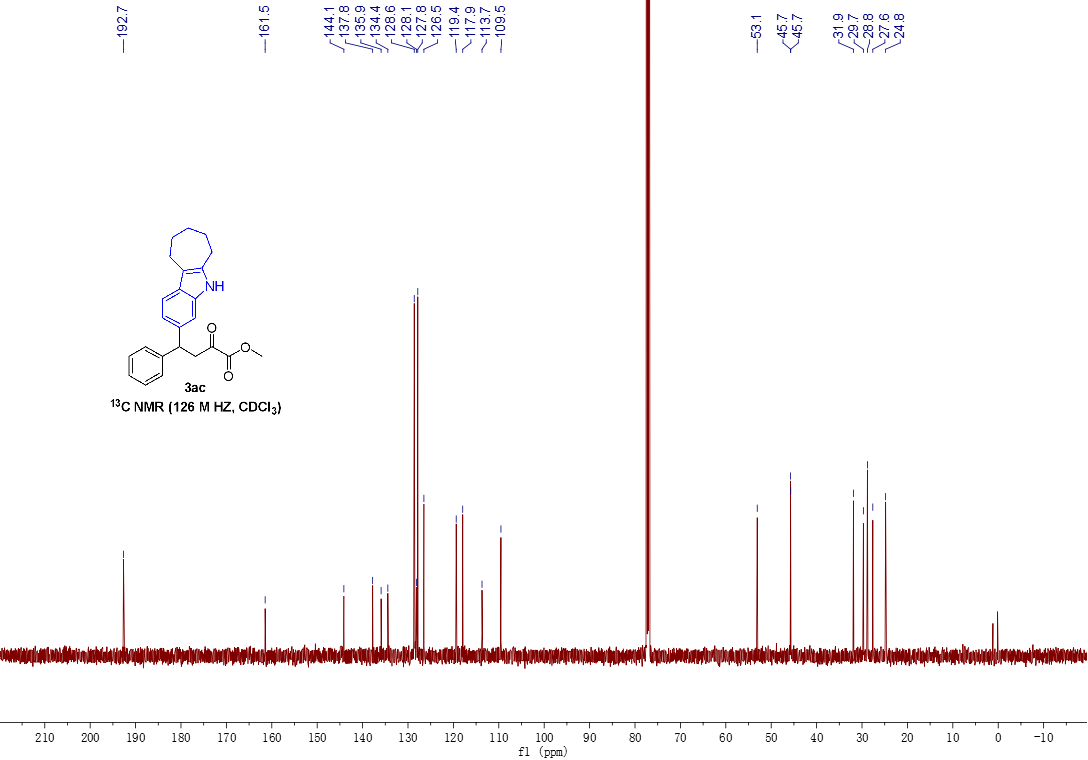
**Figure S39. ^13^C NMR (126 MHz, CDCl_3_) spectrum of 3ac**


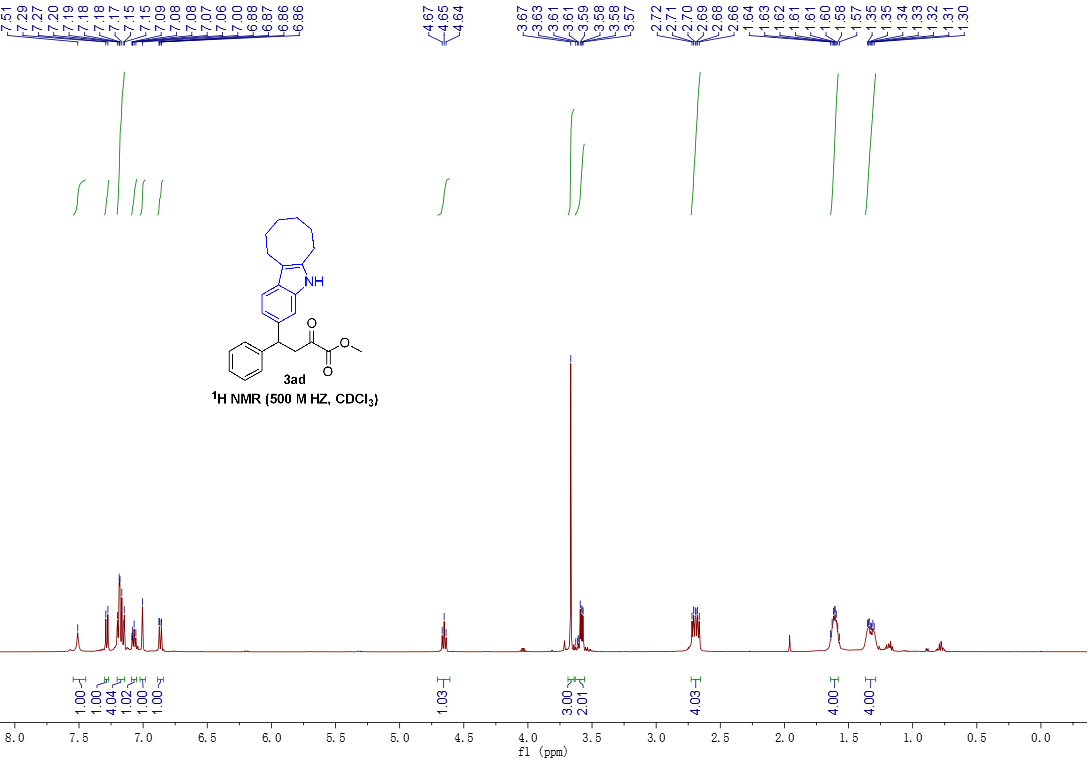
**Figure S40. ^1^H NMR (500 MHz, CDCl_3_) spectrum of 3ad**


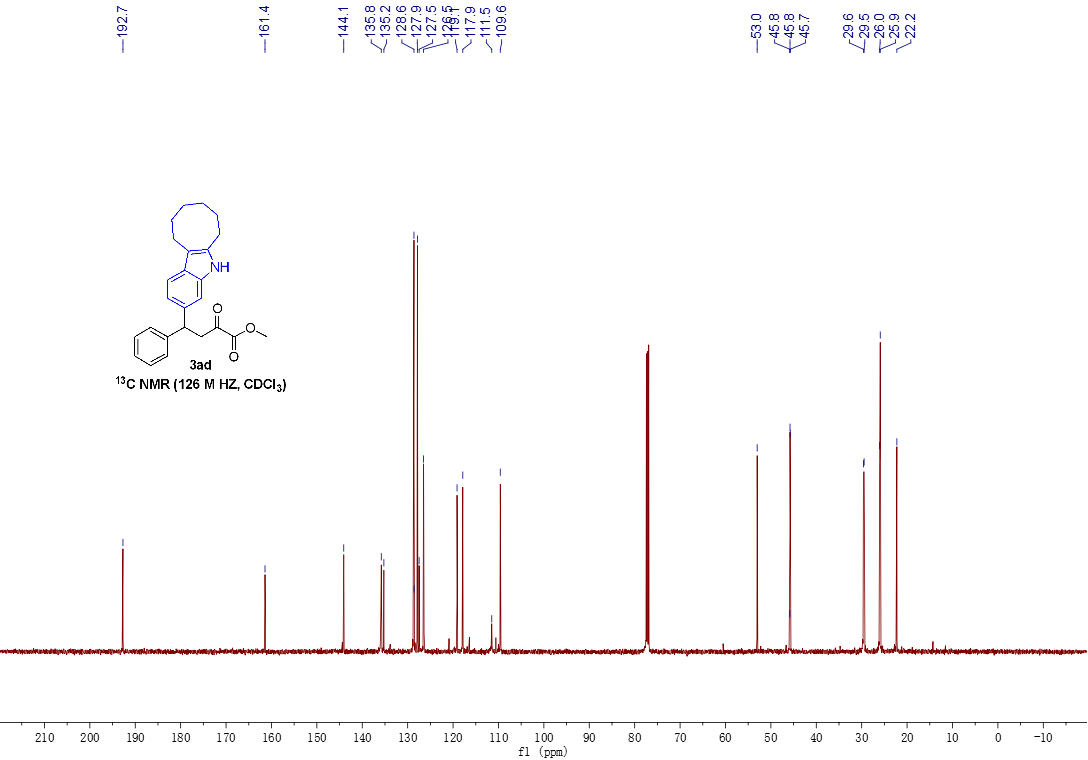
**Figure S41. ^13^C NMR (126 MHz, CDCl_3_) spectrum of 3ad**


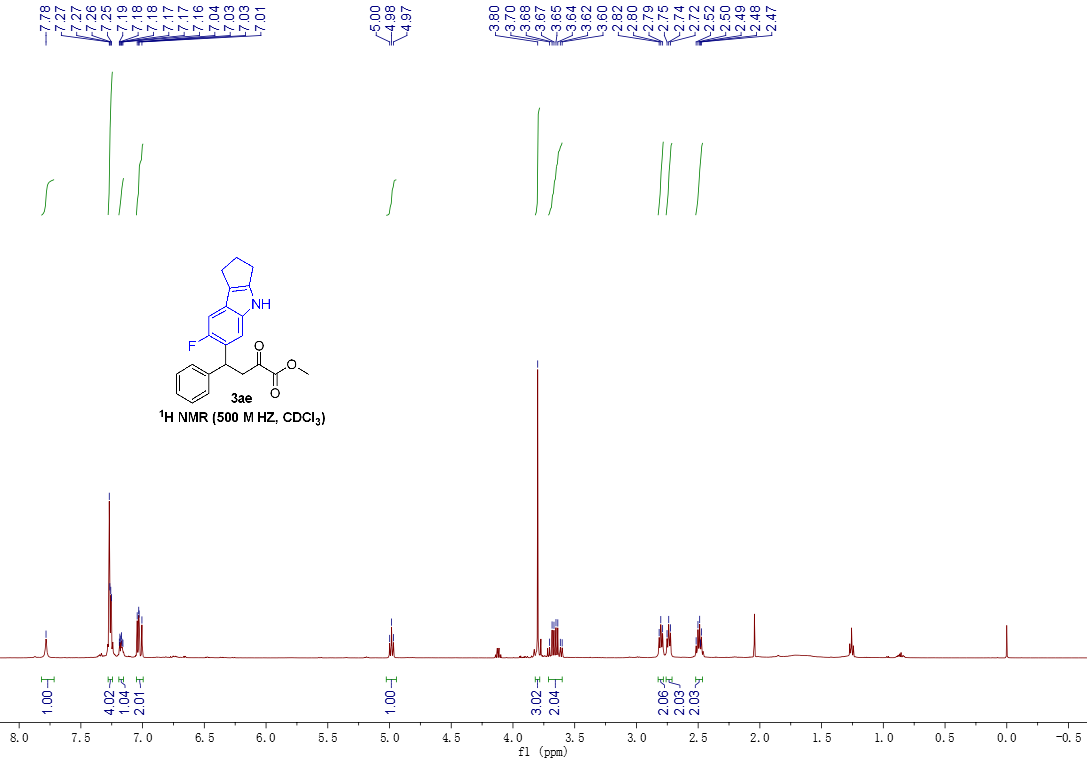
**Figure S42. ^1^H NMR (500 MHz, CDCl_3_) spectrum of 3ae**


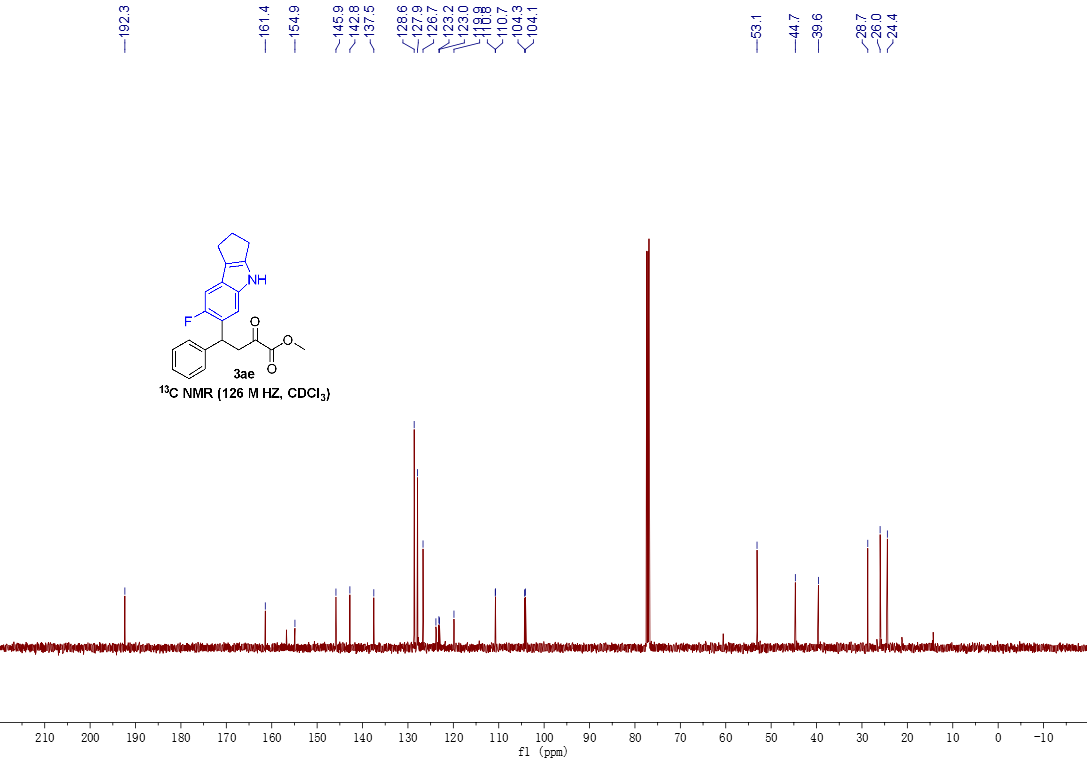


**Figure S43. ^13^C NMR (126 MHz, CDCl_3_) spectrum of 3ae**


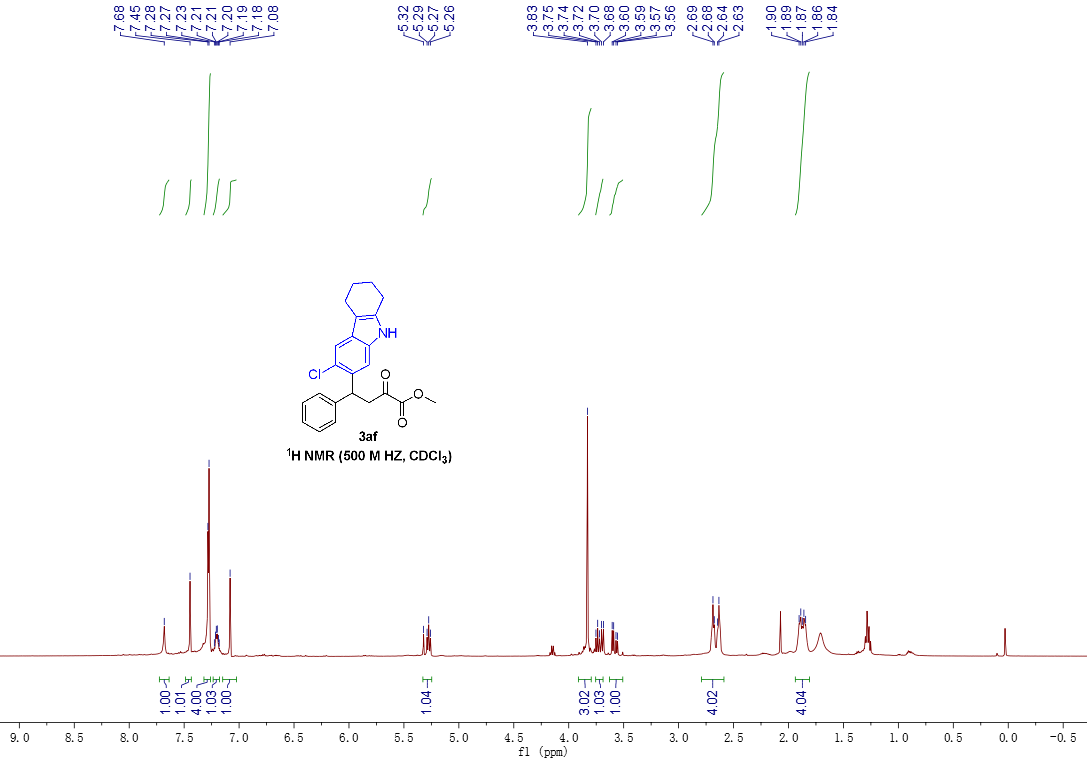


**Figure S44. ^1^H NMR (500 MHz, CDCl_3_) spectrum of 3af**


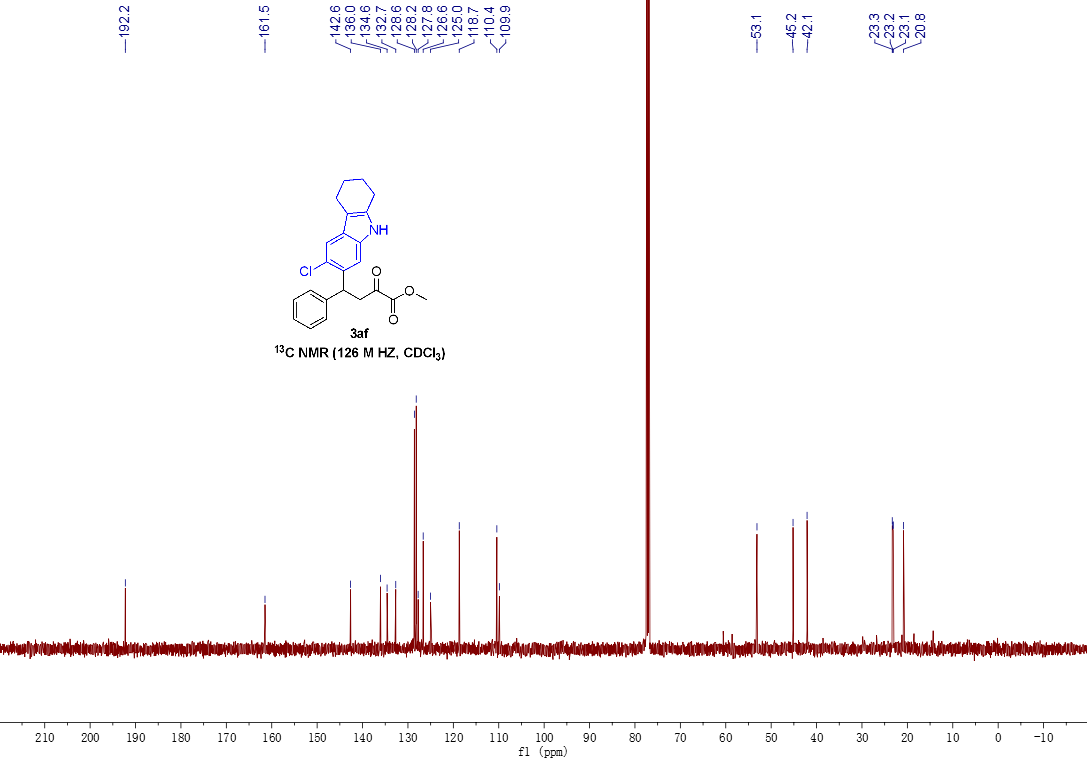


**Figure S45. ^13^C NMR (126 MHz, CDCl_3_) spectrum of 3af**


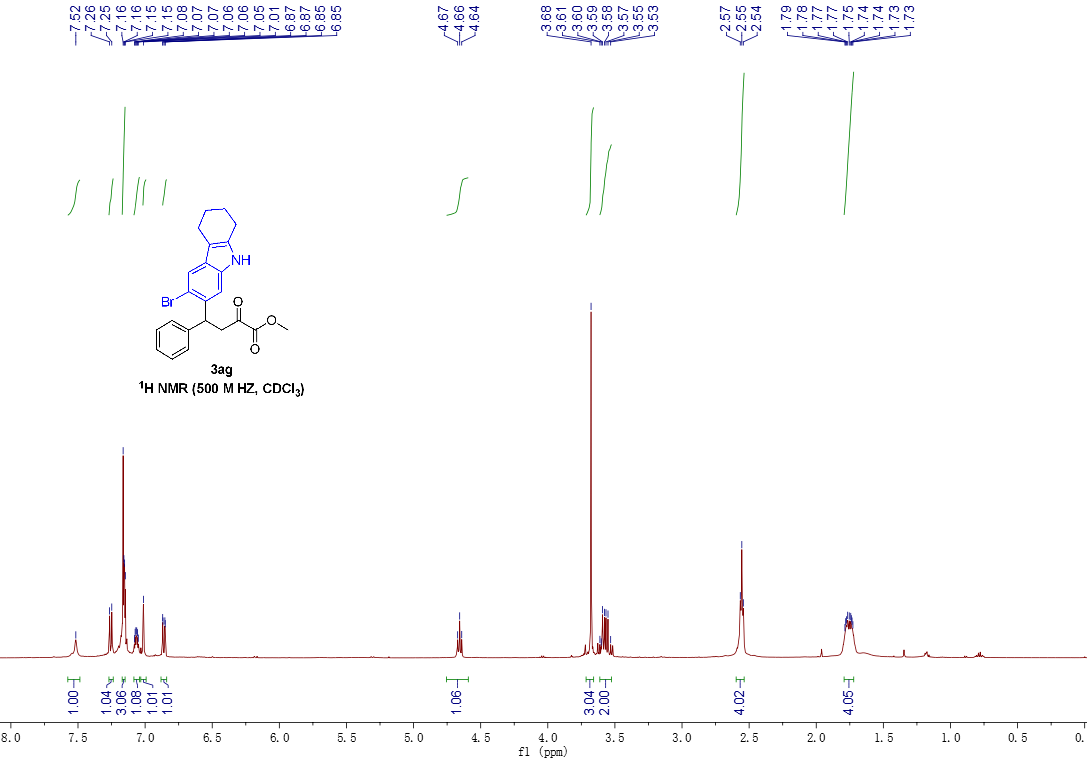
**Figure S46. ^1^H NMR (500 MHz, CDCl_3_) spectrum of 3ag**


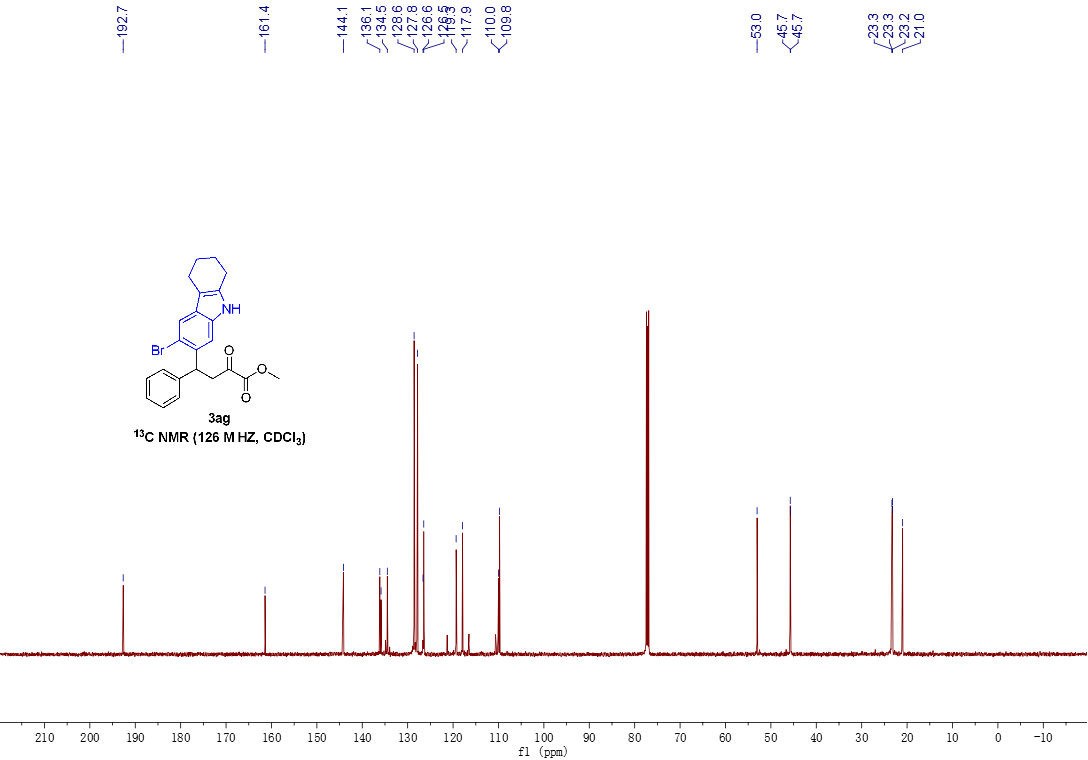


**Figure S47. ^13^C NMR (126 MHz, CDCl_3_) spectrum of 3ag**


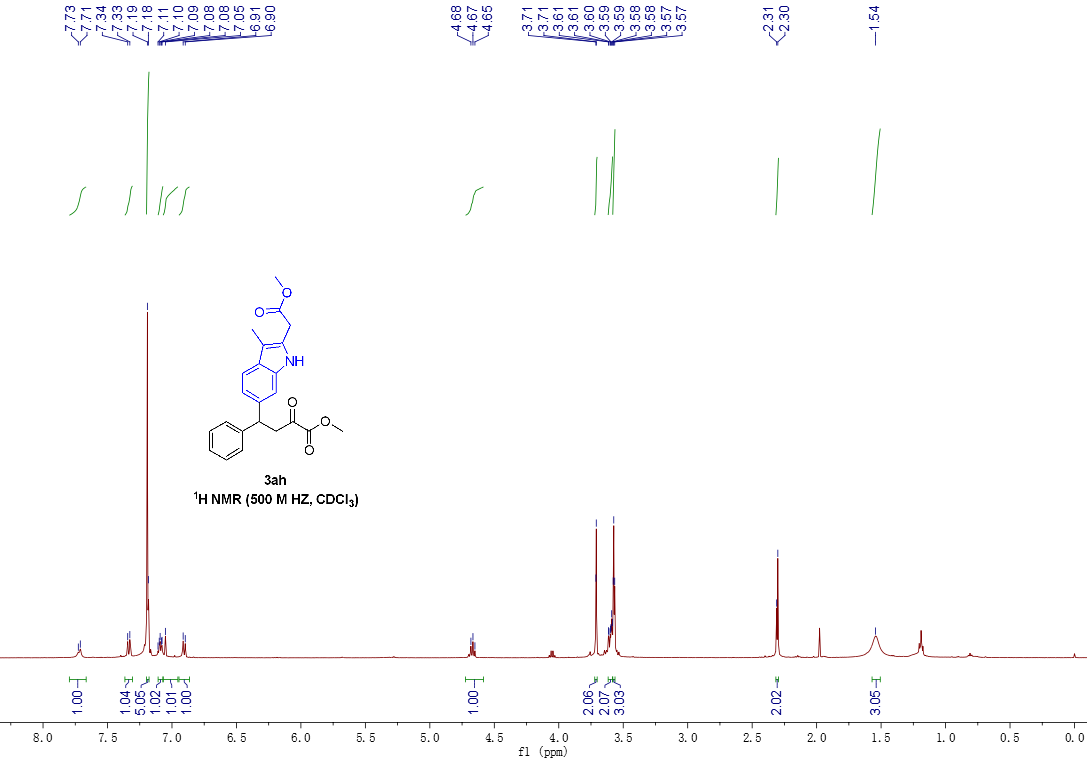
**Figure S48. ^1^H NMR (500 MHz, CDCl_3_) spectrum of 3ah**


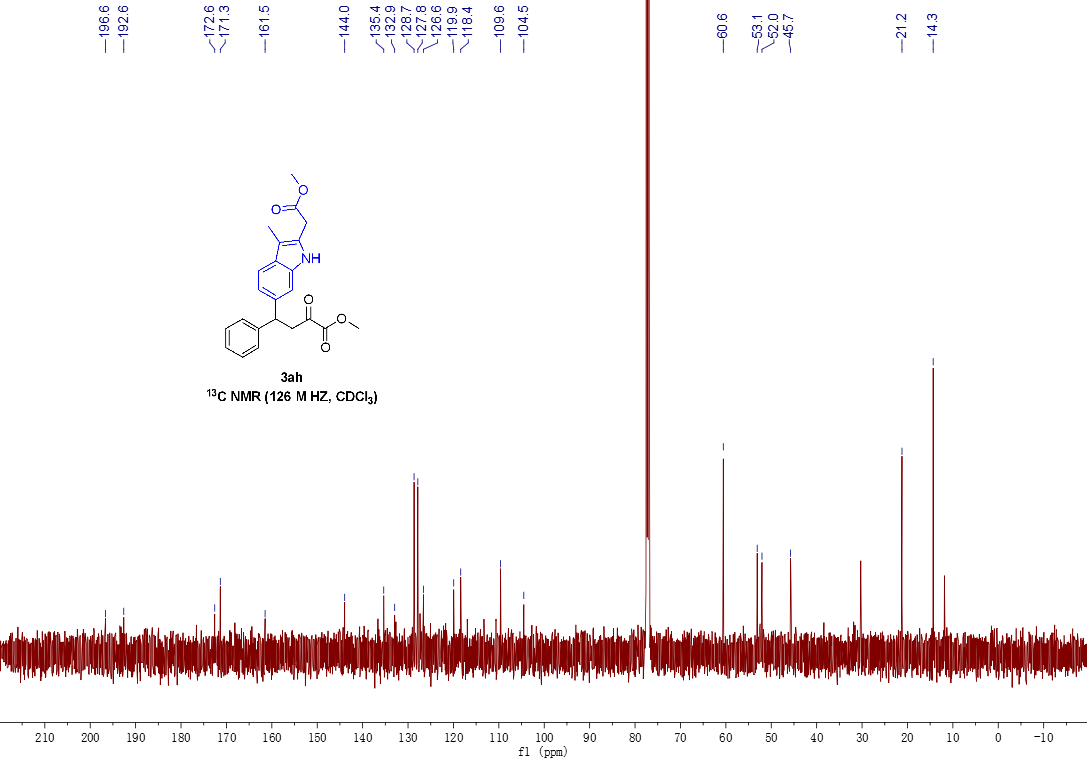
**Figure S49. ^13^C NMR (126 MHz, CDCl_3_) spectrum of 3ah**


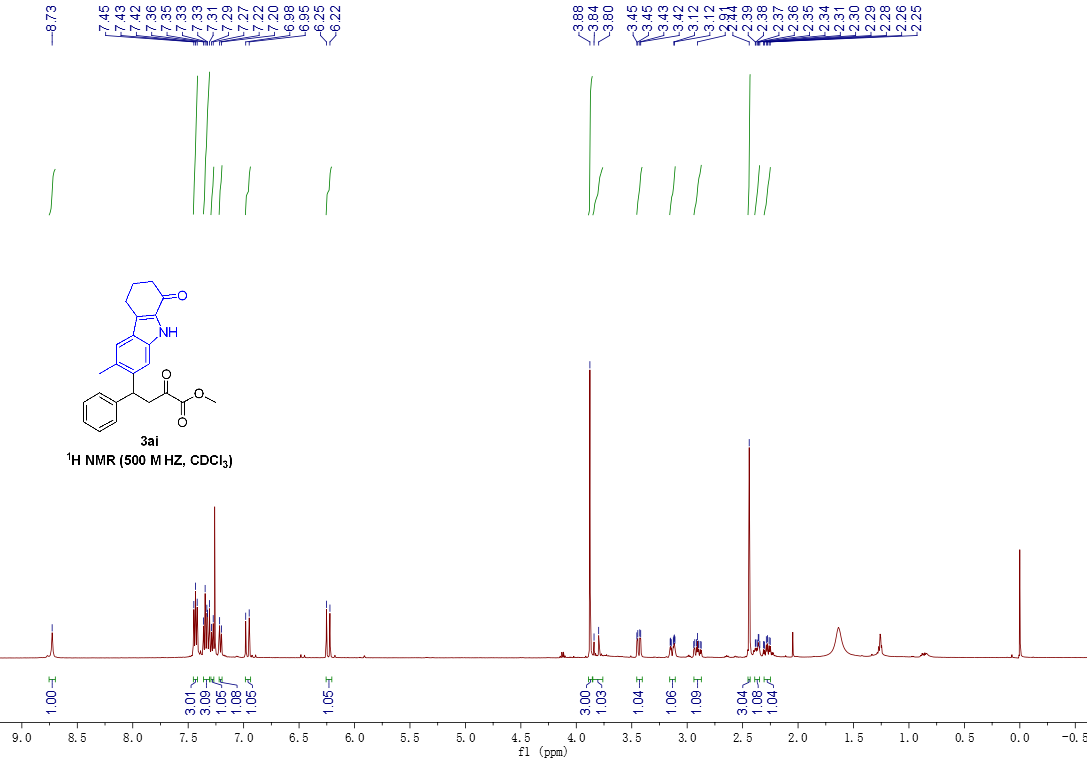


**Figure 50. ^1^H NMR (500 MHz, CDCl_3_) spectrum of 3ai**


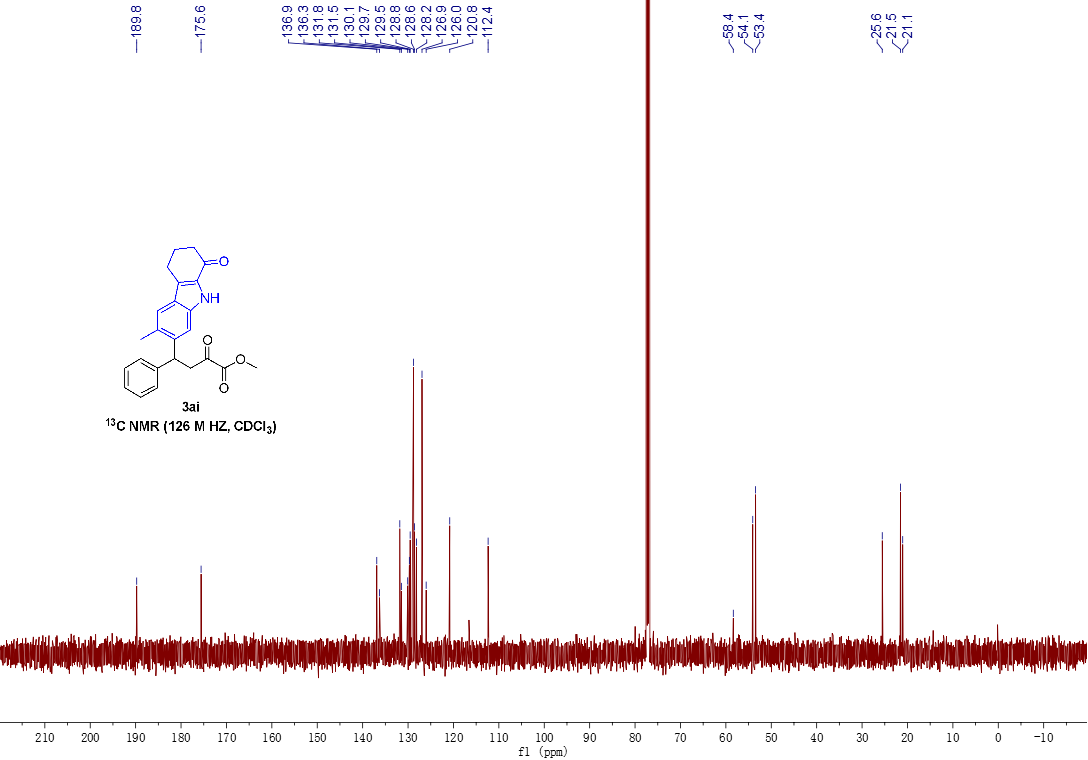


**Figure S51. ^13^C NMR (126 MHz, CDCl_3_) spectrum of 3ai**
